# Orchestration of *S. aureus* EV biogenesis by nutrient availability through quorum sensing

**DOI:** 10.1128/mbio.01598-26

**Published:** 2026-10-12

**Authors:** Clariss Limso Yamamoto, Meta Kuehn

**Affiliations:** 1 Department of Biochemistry, Duke University Medical Center 609772, Durham, North Carolina, USA; University of Illinois Chicago, Chicago, Illinois, USA

**Keywords:** extracellular vesicles, *Staphylococcus aureus*, stress response

## Abstract

The release of extracellular vesicles (EVs) is a universally conserved process. Bacterial EVs package diverse cargo, including proteins and nucleic acids, and influence bacterial adaptation and survival, as well as host-pathogen interactions. Currently, our understanding of the mechanisms underlying global principles of EV biogenesis and release in *Staphylococcus aureus* and in gram-positive species, in general, is limited. Here, we describe a moderately high-throughput approach to analyze the Nebraska Transposon Mutant Library to broadly identify determinants of *agr*-dependent EV production in *S. aureus*. We show that the *agr* quorum-sensing system dictates EV production in response to nutrient availability, likely through communication with the adaptive stress response. This study demonstrates the contribution of nutritional stress to EV biogenesis and supports a conserved communication strategy that allows metabolic state to influence EV production.

**IMPORTANCE:**

Studies have demonstrated a role for gram-positive extracellular vesicles (EVs) in virulence and adaptation; however, mechanistic knowledge of EV biogenesis remains limited. Here, using a screening approach, we demonstrate how the downstream targets of the *agr*-quorum-sensing system in *Staphylococcus aureus*, α-PSMs and RNAIII, differentially contribute to and alter EV biogenesis. We show a likely conserved communication strategy that allows nutrient availability to influence EV production. Additionally, we identified numerous candidate genes potentially involved in EV biogenesis and its regulation, providing a valuable resource for future mechanistic studies.

## INTRODUCTION

Extracellular vesicles (EVs) are membrane-derived proteoliposomal nanoparticles that have been found to be produced in all three domains of life (1–3). Bacterial EVs are derived from the outermost membrane of the cell and contain a heterogeneous mixture of cellular components, including lipids, carbohydrates, lipoproteins, soluble and membrane proteins, virulence factors, toxins, nucleic acids, and metabolites, and have been shown to influence bacterial adaptation and survival as well as host-pathogen interactions.

In comparison to over 50 years of research into outer membrane vesicles (OMVs), EVs originating from gram-negative organisms, it was only within the last two decades that researchers have begun to more deeply characterize EVs from gram-positive species (also termed membrane vesicles and cytoplasmic membrane vesicles) (4, 5). In 2009, Lee and colleagues (4) reported on the proteomic composition of EVs purified from the cultures of gram-positive bacterium *Staphylococcus aureus*, confirming the secretion of EVs by gram-positive bacteria. Subsequent studies have now identified EV production to occur in various gram-positive bacteria, such as *Streptococcus pyogenes*, *Bacillus subtilis*, *Listeria monocytogenes*, and *Streptococcus pneumoniae,* to name a few (6–8).

Proteomic analyses of EVs from various gram-positive bacteria (which will be referred to as “EVs” in this study) have revealed that cytoplasmic proteins, virulence factors, and toxins comprise a large portion of the EV protein cargo (6, 7, 9, 10). It is still unknown to what degree protein cargo is sorted and loaded into EVs; however, enrichment and depletion of specific proteins in EVs compared to the parent cell have been observed, suggesting the existence of sorting mechanisms that package EV cargo (6, 11–13).

The functions of EVs are largely dependent on their cargo, and the presence of toxins, siderophores, immune evasion proteins, adhesins, and antibiotic resistance proteins clearly indicates a role for EVs in virulence. In *S. aureus*, several reports have demonstrated the immunostimulatory properties of EVs on mammalian host cells (14–17). It has also been suggested that EV-associated toxins are more potent than the soluble secreted form of the same toxin. In *S. aureus*, EV-associated α-hemolysin, but not soluble α-hemolysin, triggered keratinocyte necrosis in keratinocytes (17).

While some aspects of gram-positive EVs have been explored and uncovered, major outstanding questions in the field concern EV biogenesis and regulation of their production (6–8). A previous study described a biogenesis and release pathway for EVs in *S. aureus* dependent on the staphylococcal alpha-type phenol-soluble modulins 1-4 (α-PSM1, α-PSM2, α-PSM3, and α-PSM4), which we will refer to here as α-PSMs collectively (18). α-PSMs are small, amphipathic, α-helical peptides that act as virulence determinants and contribute to *S. aureus* pathogenesis (19–21). Wang and colleagues (19) showed that α-PSMs can promote vesicle formation, presumably by disruption of the cytoplasmic membrane due to their surfactant-like activity. Schlatterer et al. (22) later showed that α-PSMs can promote vesicle formation by increasing membrane fluidity. Aside from α-PSMs, other factors such as peptidoglycan crosslinking and permeability, wall teichoic acids (WTAs), and prophage induction have been suggested to affect EV production (18, 23).

A virulence-associated genetic pathway has also been implicated in *S. aureus* EV production. *S. aureus* mutants lacking AgrA were reported to yield undetectable levels of EVs (24). AgrA is a component of the quorum-sensing (QS) accessory gene regulator (*agr*) system, which controls a large arsenal of virulence factors, including α-PSMs (25, 26). AgrA, the response regulator of the two-component system (TCS) AgrAC, specifically upregulates *psm*α transcription by binding directly to its promoter domain (25, 27). Recently, delta‐hemolysin (*hld*), whose transcript is embedded within the *agr*-regulated small RNA, RNAIII, was demonstrated to modulate EV biogenesis (vesiculogenesis) and influence the properties of *S. aureus* EVs (28, 29). Similar to the virulence-associated TCS control of EV biogenesis in *S. aureus*, the virulence-associated group A *Streptococcus* TCS, CovRS, has been implicated in *S. pyogenes* EV production, strongly suggesting a relationship between virulence and EV production (11).

In *S. aureus*, the general stress response has also been linked to EV production. A point mutation in the alternative sigma factor B (σ^B^), which modulates the response to adaptation to environmental stresses like heat, osmotic shifts, and antimicrobials in several gram-positive bacteria, resulted in an increase in vesicle production in *S. aureus* (30, 31). A follow-up study found that the σ^B^ point mutation hindered the ability of σ^B^ to bind to the *asp23* gene promoter, resulting in decreased expression of the alkaline shock protein 23 (Asp23), a protein that is essential for cell wall stress response signaling, which consequently increased vesicle production (32). Accordingly, it seems that the general stress response and the stress response transcription regulator, σ^B^, have regulatory roles in EV biogenesis.

Both σ^B^ and AgrA are global regulators that control the expression of numerous genes (27). Consequently, their involvement in the production of EVs suggests that vesiculogenesis is not dependent on a small number of genes—instead, an as-yet undefined complex global gene network may be involved.

In this study, we aimed to expand our understanding of α-PSM/*agr*-dependent EV production using an unbiased phenotypic screening approach to broadly identify processes, pathways, and/or genetic determinants of EV production in *S. aureus*. Traditional methods of vesicle isolation to assess bacterial EV production are laborious and time-consuming, hindering high-throughput global approaches, such as the analysis of transposon mutant libraries (33, 34). Previously, a moderately high-throughput method to assess outer membrane vesicle production by gram-negative bacteria was successfully conducted to screen *Escherichia coli* transposon mutants and the non-essential Keio mutant strain library, yielding novel insights into OMV biogenesis and regulation (35, 36). Here, we describe the development of a method to assess vesicle production in all non-essential mutant *S. aureus* strains in the Nebraska Transposon Mutant Library (NTML) (37). We demonstrate that this moderately high-throughput screen can successfully identify mutants with vesiculation phenotypes by verification using the traditional ultracentrifugation method of EV isolation. We also show that EVs can aid in adaptation and survival during stress. Additionally, our results demonstrate that *S. aureus* EV biogenesis is largely orchestrated through the *agr* QS system, through RNAIII, and may be, in part, upregulated in response to nutrient limitation. Finally, our results suggest that EV biogenesis may be governed by crosstalk between several cellular processes, including the quorum sensing system and the stringent response, suggesting a possible universally conserved communication strategy that allows metabolic state to influence vesiculogenesis.

## RESULTS

### *agr*-based regulation of EV production

**Fig 1:**
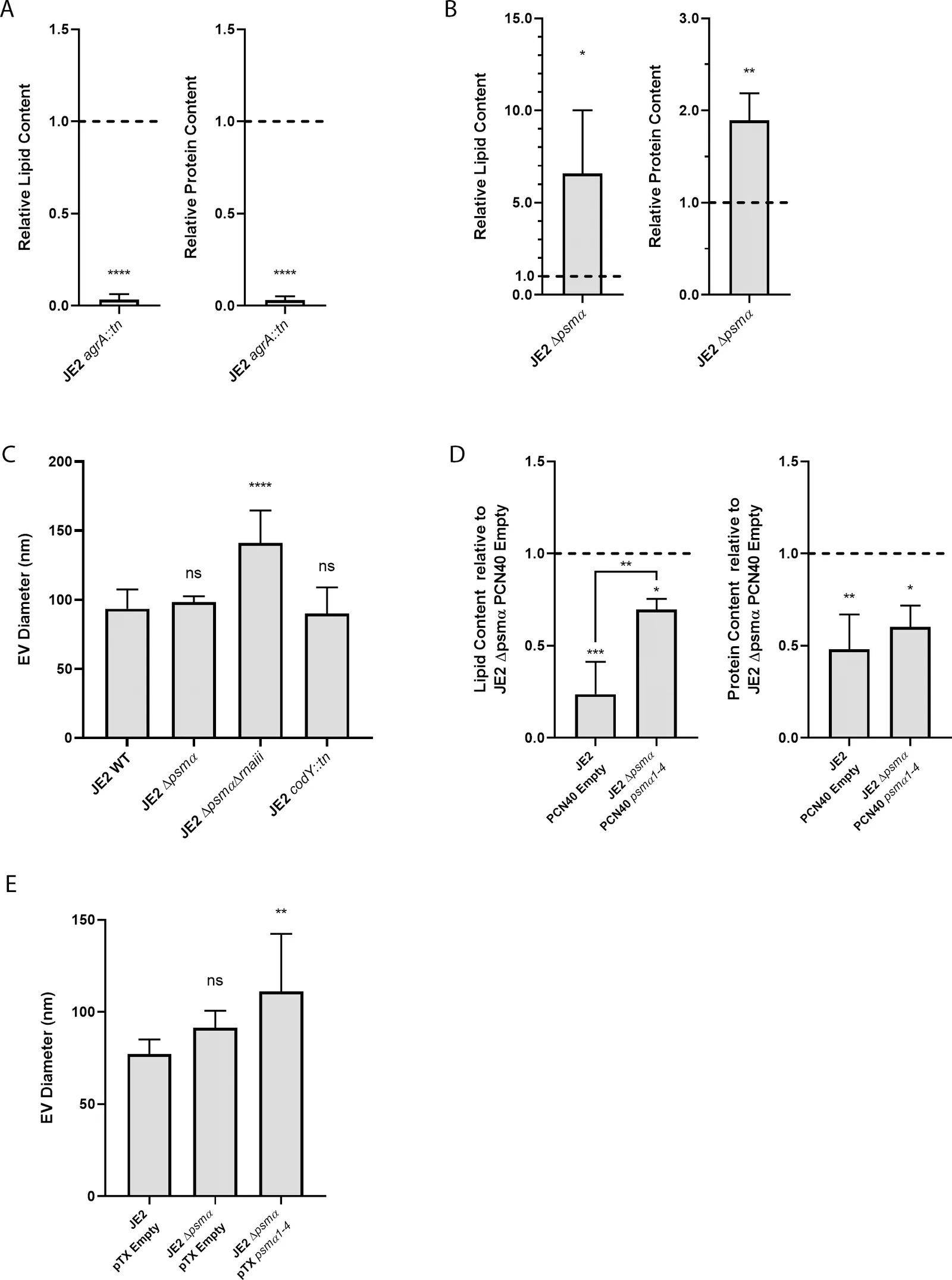
α-PSMs inhibit EV biogenesis Vesiculation values for (**A**) *agrA::tn* and (**B**) JE2 Δ*psm*α compared with those of the JE2 wild type strain represented by the horizontal dashed lines. (**C**) Average diameter of EVs from JE2 WT, Δ*psm*α, Δ*psm*αΔ*rnaiii*, and *codY::tn*, as analyzed by DLS. (**D**) Vesiculation values of JE2 Δ*psm*α complemented with the PCN40 expression vector expressing α-PSMs1-4, compared with those of JE2 Δ*psm*α expressing the empty PCN40 expression vector, represented by the horizontal dashed lines. (**E**) Average diameter of EVs from JE2 WT and Δ*psm*α expressing the empty pTX overexpression vector, and JE2 Δ*psm*α overexpressing α-PSMs1-4, as analyzed by DLS. Vesiculation values are presented as means ± SD from three independent experiments normalized to the control. Samples were compared using an unpaired *t*-test (**A and B**) or one-way ANOVA with Tukey’s multiple-comparisons test (**D**). Average EV diameters are presented as means ± SD, (*n* = 10) and were analyzed using one-way ANOVA with Dunnett's multiple-comparisons test (**C and E**). *P < 0.05, **P < 0.01, ***P < 0.001, ****P < 0.0001; ns, *P* > 0.05.

The *agr* quorum-sensing system appears to play a key role in EV biogenesis, as it has been previously shown that the *agr* mutant is severely hypovesiculating, and α-PSMs, direct targets of AgrA, have been suggested to promote EV biogenesis by acting on the cell membranes through their surfactant-like activity (15, 18, 22, 24, 38, 39). We isolated EVs from the *agrA::tn* mutant from the NTML and assessed vesicle production normalized by culture growth (OD_600_) using lipid and protein measurements by FM4-64 and Bradford assays, respectively, and compared those to that of the wild-type (WT) parent strain, JE2. The lipophilic FM4-64 dye exhibits low fluorescence in aqueous environments but fluoresces intensely in a lipophilic environment and is regularly used to stain EVs for quantification of lipid content. Just as in previous studies, we observed a hypovesiculating phenotype for the *agrA::tn* mutant (Fig. 1A) (24). Using homologous recombination, we generated the α-PSM deletion mutant (JE2 ∆*psm*α) and assessed its vesicle production (see Fig. S1 at https://github.com/clyama/Supplement-for-Yamamoto-and-Kuehn-2026-mBio01598-26). Contrary to previous studies, we observed the JE2 ∆*psm*α mutant having a hypervesiculating phenotype (Fig. 1B). In order to exclude the possibility that the phenotype we observed is due to the production of larger vesicles instead of an increase in the number of vesicles, we used dynamic light scattering (DLS) to measure the distribution and size of the vesicles produced by the ∆*psm*α mutant and the WT strain and saw no difference in the size of the EVs they produced, suggesting that the absence of α-PSMs indeed increases vesicle production (Fig. 1C; see Fig. S2 at https://github.com/clyama/Supplement-for-Yamamoto-and-Kuehn-2026-mBio01598-26). Complementation with a plasmid constitutively expressing α-PSMs reduced EV production (Fig. 1D), further confirming our findings, which support a new model in which α-PSM expression negatively, rather than positively, influences EV production in JE2. Interestingly, overexpression of α-PSMs resulted in an increase in average vesicle size, despite decreasing vesicle production as measured by protein and lipid measurements, further suggesting the negative effect of α-PSMs on EV formation (Fig. 1E; see Fig. S3 at https://github.com/clyama/Supplement-for-Yamamoto-and-Kuehn-2026-mBio01598-26).

We speculated that the effect of α-PSMs on EV biogenesis may be strain specific. Although the NTML parent strain JE2 is also a USA300 strain, JE2 has been cured of its plasmids, and we cannot disregard that difference (37). Thus, we acquired the α-PSM mutant and parent strain, as well as the complemented strains, used in the prior studies (18, 22, 38). Just as in JE2, the USA300 LAC ∆*psm*α mutant is hypervesiculating, and complementation with α-PSMs decreases EV production (see Fig. S4 at https://github.com/clyama/Supplement-for-Yamamoto-and-Kuehn-2026-mBio01598-26).

**Fig 2:**
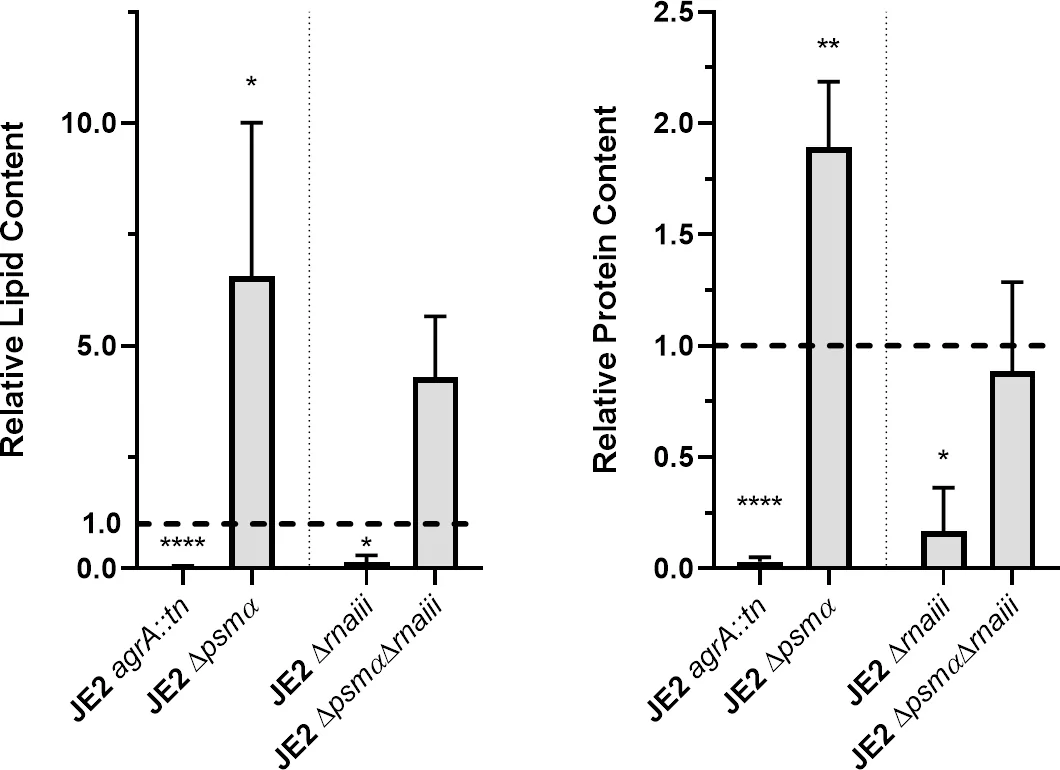
RNAIII and α-PSMs have opposing contributions to EV biogenesis. Vesiculation values for JE2 Δ*rnaiii* and Δ*psm*αΔ*rnaiii* compared to the JE2 wild-type strain, represented by the horizontal dashed lines. JE2 *agrA::tn* and Δ*psm*α mutants on the left are those presented in Fig. 1 and were added for comparison; they were not included in the statistical analyses. Values are presented as means from three independent experiments ± SD. Samples were compared using one-way ANOVA with Dunnett’s multiple-comparison test. *P < 0.05, **P < 0.01, ***P < 0.001, ****P < 0.0001.

The *agr* system regulates the expression of many genes, including key virulence factors such as proteases and toxins, through AgrA and the small RNA, RNAIII (26). In order to look into *agr*-dependent EV production further, we generated the *rnaiii* deletion mutant (JE2 ∆*rnaiii*) as well as the *psm*α and *rnaiii* deletion mutant (JE2 ∆*psm*α∆*rnaiii*) by allelic exchange and assessed their vesicle production (see Fig. S1 at https://github.com/clyama/Supplement-for-Yamamoto-and-Kuehn-2026-mBio01598-26). Deletion of *rnaiii* resulted in a hypovesiculating phenotype, suggesting that RNAIII expression promotes vesiculogenesis (Fig. 2). RNAIII and α-PSMs are direct targets of AgrA; however, deletion of *rnaiii* results in a hypovesiculating phenotype phenocopying the *agr* mutant, while the *psm*α deletion resulted in the complete opposite phenotype (Fig. 2). Intriguingly, deletion of both *rnaiii* and *psm*α resulted in an intermediate phenotype, suggesting that the effects of RNAIII and α-PSMs on EV biogenesis are independent of each other and that EV biogenesis through the *agr* quorum-sensing system is facilitated through RNAIII, rather than α-PSMs (Fig. 2).

Due to the conflicting results regarding the effect of α-PSM expression on *S. aureus* EV production observed here with those observed in previous studies, we decided to further investigate *agr*-dependent EV formation in *S. aureus*.

### Vesiculation screen development

The *agr* QS system is a global regulatory system that affects the expression of many genes involved in various cellular processes and pathways; thus, it is difficult to narrow down specific determinants of *agr*-dependent EV formation (26). In order to gain global insight into *agr*-dependent EV production, we used an unbiased phenotypic screening approach to broadly identify processes, pathways, and/or genetic determinants of EV production in *S. aureus*. The existing methods to measure gram-positive bacterial vesiculation levels are based on large-scale and time-consuming assays that are not suitable for genome-wide studies of vesiculation phenotypes (33, 34). However, previous success based on a 96-well assay to detect vesiculation phenotypes of *E. coli* mutant libraries revealed genes critical for OMV biogenesis and regulation for gram-negative bacteria (35, 36). Therefore, we designed experimental and analytical methods to assess vesicle production in small-volume *S. aureus* cultures in a moderately high-throughput, cost-effective, and reproducible fashion.

**Fig 3:**
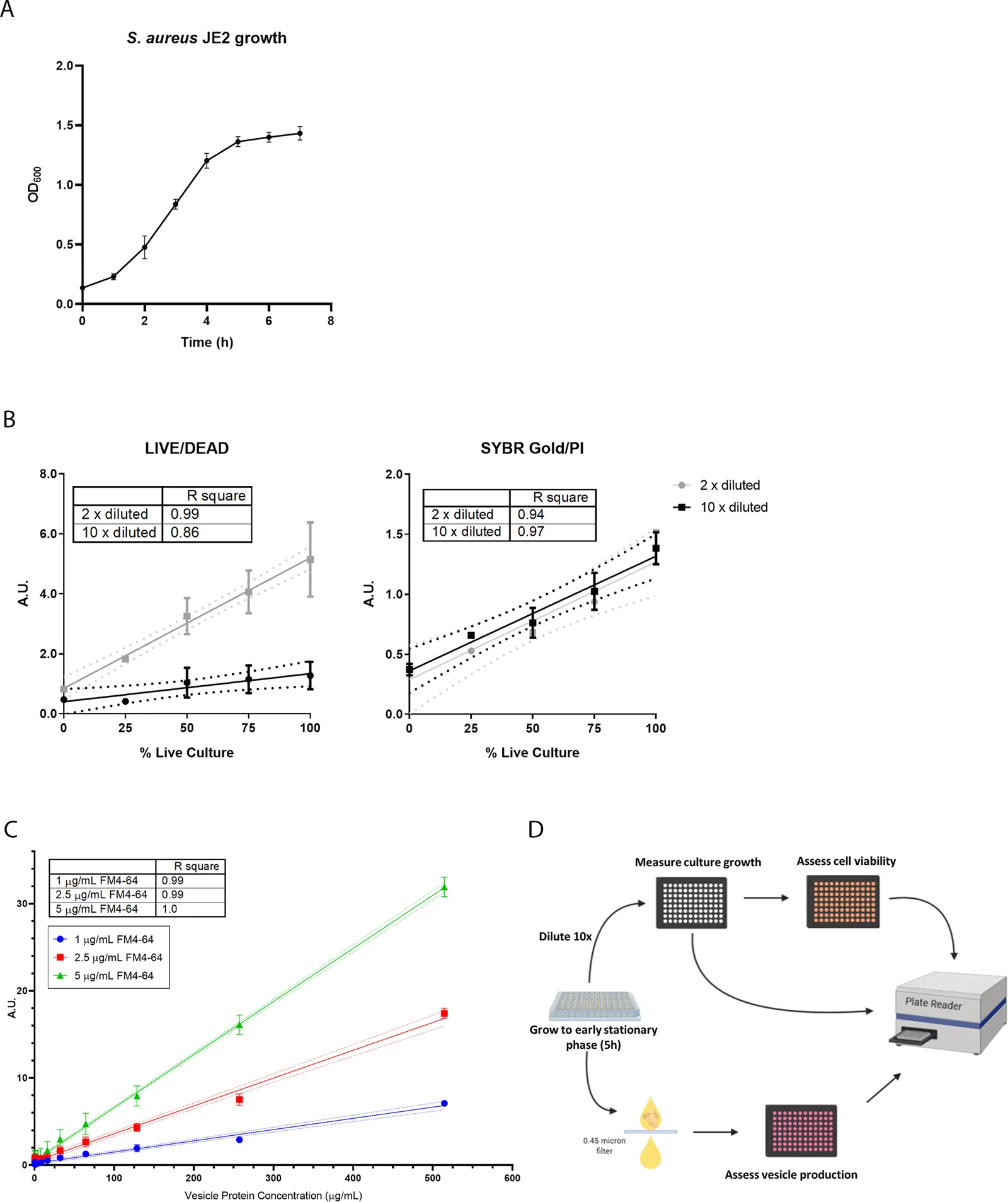
Development of the moderately high-throughput vesiculation screen (**A**) *S. aureus* JE2 growth curve in 96-well microtiter plates. (**B**) Comparison of LIVE/DEAD assay with SYBR Gold/PI viability assay in 2-fold or 10-fold-diluted mixture of cultures with varying ratios of live and dead cell cultures. Fluorescence was monitored by excitation at 488nm and emission at 500 nm for SYTO9 (LIVE), 635 nm for propidium iodide (PI; DEAD), and 535 nm for SYBR Gold. Least-squares regression analysis was performed, and dashed lines represent the 95% confidence bands for the best fit line. *R*^*2*^ values were determined as a measure goodness of fit. (**C**) Determination of FM4-64 sensitivity for detecting EVs. Purified *S. aureus* JE2 EVs were diluted with TSB to varying concentrations, as determined by protein content. FM4-64 concentrations of 1 μg/mL, 2.5 μg/mL, and 5 μg/mL were used to detect the lipid content of EVs. FM4-64 fluorescence was measured by excitation at 506 nm and emission at 750 nm. Least-squares regression analysis was performed, and dashed lines represent the 95% confidence bands for the best-fit line. *R*^*2*^ values were determined as a measure of goodness of fit. Averages from three independent experiments are presented as mean ± SD (**A–C**). (**D**) Graphic summary of the developed screen. Overnight NTML mutants were diluted 1:40 and grown for 5 h to early stationary phase. Cell culture growth was measured by OD_600_, cell viability by the SYBR Gold/PI viability assay, and vesiculation by FM4-64.

Key parameters needed to be established in the initial design of the screen included the timing of phenotype assessment during culture growth and the identification of mutants that have membrane integrity defects in the culture conditions. To decide on a time point for the screen, we relied on two considerations: first, late exponential phase cultures exhibit high viability and have had time to produce a quantifiable amount of EVs; and second, prior studies have shown that EV production in *S. aureus* is partly regulated by the *agr* quorum-sensing system and that maximal *agr* activity occurs during exponential growth, followed by a sharp drop during stationary phase (24, 40). Therefore, we chose late exponential/early stationary phase as a time point and needed to determine when cultures grown in the screening conditions would attain this phase. A 1:40 dilution of *S. aureus* JE2 overnight culture into fresh tryptic soy broth (TSB) in 96-well microtiter plates resulted in very reproducible growth, with the late exponential/early stationary phase consistently occurring at around 5 h of growth (Fig. 3A). Thus, we chose to assess vesicle production of *S. aureus* cultures after 5 h of growth. In order to rule out apparent high vesiculation values due to cell lysis, a separate assay to determine the membrane integrity of cells was necessary. Previously, a rapid 96-well assay, more sensitive than the commercially available LIVE/DEAD BacLight assay, had been developed for assessing the viability of *Borrelia burgdorferi* in a high-throughput format (41). In this assay, the ratio of viable and compromised cells is determined using membrane-permeant (SYBR Green) and non-permeant (propidium iodide [PI]) nucleic acid dyes. We compared the conventional LIVE/DEAD BacLight assay to a modified SYBR Green/PI viability assay, in which SYBR Green was substituted with a similar nucleic acid dye, SYBR Gold. *S. aureus* JE2 was grown to the early stationary phase and assigned to represent a 100% viable culture. An aliquot of the culture was heat-killed by boiling for 20 min and assigned to represent a 0% viable culture. The 100% and 0% viable cultures were then mixed in various ratios, diluted by 2- and 10-fold before being assessed for viability using either the LIVE/DEAD assay or the modified SYBR Gold/PI assay. In the samples diluted twofold, both viability assays showed a linear relationship between the signal and ratio of live to dead cells, with the *R*^2^ values of the lines of best fit being 0.99 for the LIVE/DEAD assay and 0.94 for the SYBR Gold/PI assay (Fig. 3B). However, the LIVE/DEAD assay lost sensitivity when the samples were diluted 10-fold (*R*^2^ = 0.86). On the other hand, the SYBR Gold/PI viability assay retained its sensitivity even in the 10-fold diluted samples (*R*^2^ = 0.97). We therefore chose the SYBR Gold/PI viability assay to identify mutants with compromised membrane integrity and exclude them from the screen results.

To assess EV production, the culture medium containing secreted extracellular materials, including EVs, was sterile-filtered through 96-well, 0.45 μm, polyvinylidene fluoride (PVDF) filter plates. FM4-64 was then used to quantify EVs in the filtered medium; however, the appropriate concentration of FM4-64 needed to detect EVs in this format had to be determined. We tested 1, 2.5, and 5 µg/mL concentrations of FM4-64 on a range (0.25–514.5 µg/mL by protein content) of dilutions of purified EVs from *S. aureus* (Fig. 3C). The FM4-64 values observed in the filtered spent media of the WT JE2 strain typically fall between 3 and 8 a.u.; thus, both 2.5 and 5 µg/mL FM4-64 can be used to effectively quantify EV production, but 2.5 µg/mL FM4-64 was selected for the screen (see Fig. S5 at https://github.com/clyama/Supplement-for-Yamamoto-and-Kuehn-2026-mBio01598-26). To adjust vesiculation values to account for any differences in growth, the FM4-64 signal was normalized to the final optical density (OD_600_) of each individual culture. Figure 3D provides a graphic summary of the overall process of the screen developed.

Mutants were considered to have significant hypo- or hypervesiculation phenotypes if (i) their cell viability value (SYBR Gold/PI) was more than 2 mean average deviations (MADs) below the mean viability value of the strains within the plate, and (ii) their vesiculation value (FM4-64) normalized to their growth (OD_600_) was outside 2 MADs from the mean normalized vesiculation value (FM4-64/OD_600_) of the strains within the plate.

### Pathway analysis of screen results

A total of 173 mutants, 100 hypervesiculating and 73 hypovesiculating, met the defined criteria for having vesiculation phenotypes. We note that 80 mutants were excluded from EV phenotype evaluations due to low viability (Tables 1 and 2; see Table S1 at https://github.com/clyama/Supplement-for-Yamamoto-and-Kuehn-2026-mBio01598-26).

**TABLE 1:** Hypervesiculating *S. aureus* mutants identified in the screen^*a*^

Gene ID	Gene symbol	Gene product	Description	Putative CodY binding site?^*b*^
SAUSA300_0007	*nnrD*		ADP-dependent NAD(P)H-hydrate dehydratase	Yes
SAUSA300_0012	*metX*	MetX	Homoserine O-acetyltransferase	Yes
SAUSA300_0044	*SAUSA300_0044*		MBL fold metallo-hydrolase	
SAUSA300_0141	*deoB*	DeoB	Phosphopentomutase	
SAUSA300_0173	*SAUSA300_0173*		Hypothetical protein	Yes
SAUSA300_0180	*lmrP*	LmrP	MFS transporter, DHA1 family, multidrug resistance protein B	
SAUSA300_0191	*ptsG (glcA*)	PtsG	PTS glucose EIICBA component	
SAUSA300_0192	*mupG*	MupG	Conserved hypothetical protein	
SAUSA300_0220	*pflB*	PflB	Formate acetyltransferase	
SAUSA300_0269	*SAUSA300_0269*		Choloylglycine hydrolase family protein	
SAUSA300_0294	*essI (esaG*)		Tandem EssD nuclease immunity protein paralog	Yes
SAUSA300_0305	*SAUSA300_0305*		Formate/nitrite transporter family protein	
SAUSA300_0352	*spbB*	SpbB	Signal peptide bypass ABC transporter ATP-binding subunit	
SAUSA300_0362	*SAUSA300_0362*		Mechanosensitive ion channel protein MscS	Yes
SAUSA300_0394	*SAUSA300_0394*		FAD/NAD(P)-binding Rossmann fold superfamily protein	Yes
SAUSA300_0422	*tlaA2*	TlaA2	TslA chaperone and lap secretion-signal protein TlaA2	
SAUSA300_0432	*SAUSA300_0432*		Sodium-dependent transporter	
SAUSA300_0458	*SAUSA300_0458*		Orn/Lys/Arg decarboxylase	
SAUSA300_0539	*ilvE*	IlvE	Branched-chain amino acid aminotransferase	Yes
SAUSA300_0582	*SAUSA300_0582*		Putative integral membrane protein	
SAUSA300_0592	*SAUSA300_0592*		Hypothetical protein	
SAUSA300_0605	*sarA*	SarA	Accessory regulator A	Yes
SAUSA300_0622	*SAUSA300_0622*		Putative membrane protein	
SAUSA300_0684	*fruB*	FruB	Fructose-1-phosphate kinase	
SAUSA300_0685	*fruA*	FruA	Fructose-specific permease	
SAUSA300_0706	*opuBA*	opuBA	Putative osmoprotectant ABC transporter ATP-binding protein	
SAUSA300_0740	*SAUSA300_0740*		Putative hydrolase	
SAUSA300_0791	*gcvH*	GcvH	Glycine cleavage system protein H	
SAUSA300_0833	*nagD*		TIGR01457 family HAD-type hydrolase	
SAUSA300_0867	*spsA*	SpsA	Signal peptidase IA	
SAUSA300_0869	*rexB (addB*)	RexB	ATP-dependent helicase/deoxyribonuclease subunit B	Yes
SAUSA300_0903	*yjbH*	YjbH	ClpXP substrate-recognition adaptor protein	
SAUSA300_0905	*SAUSA300_0905*		Hypothetical protein	Yes
SAUSA300_0918	*ypfP (ugtP*)	YpfP (UgtP)	Diacylglycerol glucosyltransferase	
SAUSA300_0924	*ktrD*	KtrD	Potassium transport and sodium transport	
SAUSA300_0946	*menD*	MenD	Inyl-6-hydroxy-2,4-cyclohexadiene-1-carboxylic acid synthase/2-oxoglutarate decarboxylase	
SAUSA300_0962	*qoxB*	QoxB	Cytochrome aa3 quinol oxidase subunit I	Yes
SAUSA300_0973	*purM*	PurM	Phosphoribosylaminoimidazole synthetase	Yes
SAUSA300_0980	*auxA*	AuxA	Auxiliary factor A	
SAUSA300_0996	*lpdA* (*pdhD*)	LpdA (PdhD)	Pyruvate dehydrogenase complex E3 lipoamide dehydrogenase	Yes
SAUSA300_1003	*auxB*	AuxB	Auxiliary factor B	Yes
SAUSA300_1020	*lpgD*	LpgD	Lysophosphatidylglycerol phospholipase D	
SAUSA300_1061	*ssl14*		Staphylococcal superantigen-like protein 14| exotoxin 3	Yes
SAUSA300_1093	*pyrB*	PyrB	Aspartate carbamoyltransferase catalytic subunit	Yes
SAUSA300_1118	*vfrA*		Asp23/Gls24 family envelope stress response protein	
SAUSA300_1139	*sucD*	SucD	Succinyl-CoA synthetase subunit alpha	
SAUSA300_1148	*codY*	CodY	Transcriptional repressor CodY	Yes
SAUSA300_1170	*SAUSA300_1170*		Transcriptional regulator, GntR family	Yes
SAUSA300_1182	*porA*	PorA	Pyruvate ferredoxin oxidoreductase, alpha subunit	
SAUSA300_1183	*porB*	PorB	2-oxoglutarate ferredoxin oxidoreductase subunit beta	
SAUSA300_1185	*miaB*	MiaB	(Dimethylallyl)adenosine tRNA methylthiotransferase	
SAUSA300_1195	*miaA*	MiaA	tRNA delta (2)-isopentenylpyrophosphate transferase	Yes
SAUSA300_1276	*opp2B (nikB*)	Opp-2B	Oligopeptide permease, channel-forming protein	
SAUSA300_1291	*dapI*	DapI	Hippurate hydrolase	Yes
SAUSA300_1352	*lhaT (ypjA*)	LhaT	Diacyl-lipoprotein heptaprenylglyceryl N-acetyltransferase LhaT	Yes
SAUSA300_1359	*hepT*	HepT	Polyprenyl synthetase	
SAUSA300_1463	*brxB*	BrxB	Bacilloredoxin	
SAUSA300_1504	*comGA*	ComGA	Putative competence protein ComGA	Yes
SAUSA300_1512	*pbp3*	Pbp3	Penicillin-binding protein 3	
SAUSA300_1531	*phoH*	PhoH	Phosphate starvation-induced protein, PhoH family	Yes
SAUSA300_1569	*trhP1* (*yrrO*)	TrhP1 (YrrO)	Anaerobic tRNA uridine (34) hydroxylation protein P1	
SAUSA300_1570	*trhP2* (*yrrN*)	TrhP2 (YrrN)	Anaerobic tRNA uridine (34) hydroxylation protein P2	
SAUSA300_1590	*relA* (*rel*)	RelA (Rel)	Bifunctional stringent response alarmone pyrophosphokinase/pyrophosphohydrolase	Yes
SAUSA300_1618	*hemX*	HemX	hemA concentration negative effector hemX	Yes
SAUSA300_1628	*lysP*	LysP	Lysine-specific permease	
SAUSA300_1698	*SAUSA300_1698*		Hypothetical protein	
SAUSA300_1710	*pldB*	PldB	Lysophospholipase	
SAUSA300_1713	*ribA (ribBA*)	ribBA/RibA	Riboflavin biosynthesis bifunctional GTP cyclohydrolase II/3,4-dihydroxy-2-butanone 4-phosphate synthase	Yes
SAUSA300_1734	*rppH*	RppH (YtkD)	Nucleoside triphosphatase YtkD	
SAUSA300_1742	*SAUSA300_1742*		Putative lipoprotein	
SAUSA300_1788	*SAUSA300_1788*		Putative secreted protein	
SAUSA300_1854	*recX*	RecX	Recombination regulator RecX	
SAUSA300_1866	*vraS*	VraS	Two-component sensor histidine kinase	
SAUSA300_1906	*SAUSA300_1906*		Hypothetical protein	
SAUSA300_1921	*sdp*	SDP	Prophage-encoded truncated SH3-domain protein	
SAUSA300_1942	*SAUSA300_1942*		Conserved hypothetical phage protein	
SAUSA300_1967	*tmcA*	TmcA	Prophage type I addiction module noncoding RNA antitoxin TmcA	
SAUSA300_1975	*lukH (lukA*)	LukH (LukA)	Aerolysin/leukocidin family protein	
SAUSA300_2030	*SAUSA300_2030*		Hypothetical membrane protein	
SAUSA300_2037	*cshA*	CshA	Degradosome RNA helicase CshA	
SAUSA300_2047	*thiE*	ThiE	Thiamine-phosphate pyrophosphorylase	
SAUSA300_2050	*tenA*	TENA/THI-4 family protein	Thiaminase II	
SAUSA300_2055	*murA* (*murA1*)	MurA	UDP-N-acetylglucosamine 1-carboxyvinyltransferase	Yes
SAUSA300_2098	*arsR* (*czrA*)	arsR/CzrA	ArsR family transcriptional regulator	
SAUSA300_2099	*czrB*	Czrb	Cation efflux family protein	
SAUSA300_2107	*mtlA* (*mtlF*)	MtlA	PTS system, mannitol-specific IIA component	
SAUSA300_2154	*lacB*	LacB	Galactose-6-phosphate isomerase subunit LacB	Yes
SAUSA300_2173	*truA*	TruA	tRNA pseudouridine synthase A	
SAUSA300_2175	*cbiO2 (ecfA2*)	CbiO	Cobalt transporter ATP-binding subunit, energy-coupling factor transporter ATPase EcfA′	
SAUSA300_2236	*ydbM*		Hypothetical protein	Yes
SAUSA300_2237	*yut*	Yut	Urea transporter	
SAUSA300_2239	*ureB*	UreB	Urease subunit beta	Yes
SAUSA300_2243	*ureG*	UreG	Urease accessory scaffold GTPase UreG	Yes
SAUSA300_2264	*rpiRc*	RpiRc	RpiR family phosphosugar-binding transcriptional regulator	
SAUSA300_2308	*hssR*	HssR	Heme sensor system response regulator	
SAUSA300_2365	*hlgA*	HlgA	Gamma-hemolysin component A	
SAUSA300_2371	*bioB*	BioB	Biotin synthase	
SAUSA300_2377	*glxK*	GlxK	Glycerate kinase	
SAUSA300_2555	*gpxA2*		Glutathione peroxidase	Yes
SAUSA300_2559	*nsaR* (*braR*)	NsaR/BraR	DNA-binding response regulator	

^*a*^ Mutants that have FM4-64/OD_600_ values >2 mean average deviations from the average FM4-64/OD_600_ of mutants on the same plate. Values are means from at least three replicates. KEGG and AureoWiki databases were used as references for gene annotation (3, 42).

^*b*^ Presence of putative CodY binding sites was determined using references 43–45.

**TABLE 2:** Hypovesiculating *S. aureus* mutants identified in the screen^*a*^

Gene ID	Gene symbol	Gene product	Description	Putative CodY binding site?^*b*^
SAUSA300_0030	*ugpQ*	UgpQ	Putative glycerophosphoryl diester phosphodiesterase	
SAUSA300_0034	*SAUSA300_0034*		IS1272mec transposase	Yes
SAUSA300_0047	*SAUSA300_0047*		Hypothetical protein	
SAUSA300_0081	*SAUSA300_0081*		Conserved hypothetical protein	Yes
SAUSA300_0108	*ohyA*	OhyA	Antimicrobial fatty acid resistance hydratase	
SAUSA300_0113	*spA*	SpA	Immunoglobulin G binding protein A precursor	
SAUSA300_0116	*sirB*	SirB	Holo-staphyloferrin B ABC transporter permease SirB	
SAUSA300_0136	*sasD*	SasD	Cell wall surface anchor family protein	
SAUSA300_0140	*deoC1*	DeoC	Deoxyribose-phosphate aldolase	
SAUSA300_0157	*cap5F*	Cap5F	Capsular polysaccharide biosynthesis protein Cap5F	Yes
SAUSA300_0187	*rocD/ArgD*	RocD	Ornithine aminotransferase, arginine biosynthesis	
SAUSA300_0188	*brnQ1*	BrnQ	Branched-chain amino acid transport system II carrier protein	Yes
SAUSA300_0247	*tarL*′ (*tarK*)	TarL′ (TarK)	Short-length ribitol-phosphate polymerase	Yes
SAUSA300_0268	*SAUSA300_0268*		Putative drug transporter	
SAUSA300_0339	*SAUSA300_0339*		LLM class flavin-dependent oxidoreductase	
SAUSA300_0431	*SAUSA300_0431*		Hypothetical protein	
SAUSA300_0481	*mfd*	Mfd	Transcription-repair coupling factor	
SAUSA300_0547	*sdrD*	SdrD	sdrD adhesin	
SAUSA300_0578	*SAUSA300_0578*		Hypothetical protein	
SAUSA300_0645	*graR*	GraR	DNA-binding response regulator	
SAUSA300_0744	*lgt*		Prolipoprotein diacylglyceryl transferase	
SAUSA300_0759	*pgm* (*gpmI*)	GpmI	Phosphoglyceromutase	Yes
SAUSA300_0789	*trxP*	TrxP	Primary sulfide-stress thioredoxin	
SAUSA300_0873	*cdr*	Cdr	Coenzyme A disulfide reductase	
SAUSA300_0900	*SAUSA300_0900*		Putative competence protein	
SAUSA300_0970	*purQ*	PurQ	Phosphoribosylformylglycinamidine synthase I	Yes
SAUSA300_1015	*ctaA*	CtaA	Cytochrome oxidase assembly protein	
SAUSA300_1059	*ssl12*	SSL12	Staphylococcal superantigen-like protein 12	Yes
SAUSA300_1090	*rluD*	RluD	Ribosomal large subunit pseudouridine synthase D	
SAUSA300_1114	*rsgA* (*yjeQ*)	RsgA	30S ribosome checkpoint protein and ribosome-associated GTPase RsgA	
SAUSA300_1203	*SAUSA300_1203*		Hypothetical protein	Yes
SAUSA300_1327	*ebh*	Ebh	Cell surface protein	Yes
SAUSA300_1425	*SAUSA300_1425*		Hypothetical phage protein	
SAUSA300_1550	*SAUSA300_1550*		SAM-dependent methyltransferase	Yes
SAUSA300_1592	*recJ*	RecJ	Single-stranded-DNA-specific exonuclease RecJ	
SAUSA300_1744	*SAUSA300_1744*		Conserved hypothetical protein	
SAUSA300_1752	*hsdM*		Type I restriction-modification system, M subunit, DNA-methyltransferase (adenine-specific)	
SAUSA300_1791	*yhaM/cbf1*	Cbf1	3′−5′ exoribonuclease YhaM	
SAUSA300_1801	*fumC*	FumC	fumarate hydratase, class II	
SAUSA300_1802	*SAUSA300_1802*		Conserved hypothetical protein	
SAUSA300_1810	*tnp3*	Tnp	IS1181, transposase	
SAUSA300_1905	*SAUSA300_1905*		Choloylglycine hydrolase	
SAUSA300_1911	*pmtC*	PmtC	Phenol-soluble modulin α export ABC transporter ATP-binding subunit	
SAUSA300_1935	*tcp* (*np*)		Serogroup F prophage tail-completion/neck protein	
SAUSA300_1936	*SAUSA300_1936*		Conserved hypothetical phage protein	
SAUSA300_1948	*SAUSA300_1948*		phi77 ORF069-like protein	
SAUSA300_1952	*SAUSA300_1952*		phiPV083 ORF027-like protein	
SAUSA300_1989	*agrB*	AgrB	Accessory gene regulator protein B	Yes
SAUSA300_2090	*deoC2 (deoC*)	DeoC	Deoxyribose-phosphate aldolase	
SAUSA300_2094	*pcdA*	PcdA	Orthogonal placement of cell division plane factor A	
SAUSA300_2095	*SAUSA300_2095*		Conserved hypothetical protein	
SAUSA300_2100	*SAUSA300_2100*		Lytic regulatory transmembrane protein	
SAUSA300_2207	*pbuG*	PbuG	Xanthine/uracil permease family protein	
SAUSA300_2211	*SAUSA300_2211*		Malonate transporter	
SAUSA300_2219	*moaA*		Cyclic pyranopterin monophosphate synthase A	
SAUSA300_2224	*moeA*	MoeA	Molybdopterin biosynthesis protein A, moco	
SAUSA300_2267	*SAUSA300_2267*		HAD superfamily hydrolase	Yes
SAUSA300_2302	*tcaA*	TcaA	Teicoplanin resistance-associated membrane protein TcaA protein	Yes
SAUSA300_2314	*SAUSA300_2314*		CDP-glycerol glycerophosphotransferase family protein	
SAUSA300_2349	*SAUSA300_2349*		Formate/nitrite transporter family protein	Yes
SAUSA300_2354	*dsbA*	DsbA	Disulfide bond donor thiol-disulfide oxidoreductase	
SAUSA300_2362	*gpmA*	GpmA	2,3-bisphosphoglycerate-dependent phosphoglycerate mutase	
SAUSA300_2391	*opuCc*	OpuCc	Carnitine-specific compatible solute ABC transporter carnitine-binding subunit	Yes
SAUSA300_2473	*SAUSA300_2473*		Conserved hypothetical protein	
SAUSA300_2475	*SAUSA300_2475*		Acyl-CoA thioester hydrolase	
SAUSA300_2500	*crtQ*		Staphyloxanthin synthesis 4,4′-diaponeurosporenoate glycosyltransferase	
SAUSA300_2503	*SsaA2*		Secretory antigen precursor SsaA ssaA2	Yes
SAUSA300_2504	*oatA*	OatA	Peptidoglycan O-acetyltransferase OatA	
SAUSA300_2521	*SAUSA300_2521*		Hypothetical protein	
SAUSA300_2539	*SAUSA300_2539*		Aminotransferase, 4-aminobutyrate aminotransferase	Yes
SAUSA300_2618	*SAUSA300_2618*		ECF-type riboflavin transporter substrate-binding protein	Yes
SAUSA300_2624	*SAUSA300_2624*		Putative membrane protein	
SAUSA300_2627	*SAUSA300_2627*		2-oxoglutarate/malate translocator	

^*a*^ Mutants that have FM4-64/OD_600_ values <2 mean average deviations from the average FM4-64/OD_600_ of mutants on the same plate. Values are means from at least three replicates. KEGG and AureoWiki databases were used as references for gene annotation (3, 42).

^*b*^ Presence of putative CodY binding sites was determined using references 43–45.

We performed a Gene Ontology (GO) analysis to determine cellular processes or pathways enriched in our data set. Because the GO database is not annotated for any USA300 strain, the strain NCTC8325 was used as the reference strain. Of the 173 gene mutations identified, only 162 are present in the strain NCTC8325. Therefore, the 11 missing genes were excluded from the GO analysis (see Table S2 at https://github.com/clyama/Supplement-for-Yamamoto-and-Kuehn-2026-mBio01598-26). The analysis identified the enrichment of genes involved in cell wall biogenesis, as expected based on prior studies (Table 3; see Table S3 at https://github.com/clyama/Supplement-for-Yamamoto-and-Kuehn-2026-mBio01598-26 for full table). The GO analysis also showed the vesiculation mutant data set to have an enrichment of genes involved in stress responses, including transcription factors and two-component systems that control the expression of numerous genes in response to stimuli, as well as genes involved in altering membrane fluidity, including branched-chain amino acid (BCAA; isoleucine, leucine, and valine) biosynthesis and lipid biogenesis genes, among others.

**TABLE 3:** GO analysis^*a*^

Pathway^*a*^	nGenes	Pathway genes	Fold enrichment
Hexose catabolic process	2	5	20.20
Transcriptional regulatory protein, C terminal	3	8	11.65
Nucleotide catabolic process	4	12	10.35
Hexose metabolic process	4	20	10.10
Glucose metabolic process	3	18	8.42
Teichoic acid biosynthetic process	3	19	7.97
Aerobic respiration	3	22	6.89
Vitamin biosynthetic process	4	32	6.31
GTP binding	5	25	6.21
Signaling	5	41	6.16
Guanyl nucleotide binding	6	31	6.01
Mostly uncharacterized, including biosynthesis of various secondary metabolites—part 3 and DNA binding HTH domain, AraC-type	6	34	5.48
Carbohydrate derivative catabolic process	5	29	5.35
Citrate cycle (TCA cycle) and branched-chain amino acid metabolic process	6	37	5.04
Mixed, including biosynthesis of various secondary metabolites—part 3 and two-component system	7	45	4.83
Peptidoglycan-based cell wall biogenesis	4	46	4.39
Ion transmembrane transport	8	94	4.30
RNA modification	4	47	4.30
Cell wall biogenesis	4	47	4.30
Cell wall organization or biogenesis	5	61	4.14
Lipid metabolic process	6	75	4.04
RNA processing	5	65	3.89
Cellular lipid metabolic process	5	66	3.83
Nucleoside monophosphate biosynthetic process and nucleobase-containing compound catabolic process	7	59	3.68
Response to stimulus	13	183	3.59
Regulation of transcription, DNA-templated	9	129	3.52
Cellular response to stimulus	9	132	3.44
Response to stress	7	103	3.43
Regulation of cellular metabolic process	10	157	3.22
Winged helix-like DNA-binding domain superfamily	9	91	3.07
Nucleoside phosphate metabolic process	11	112	3.05
Transmembrane transport	22	291	2.35
Purine ribonucleoside triphosphate binding	20	311	2.00

^*a*^ GO Analysis was performed using the ShinyGO version 0.85 database (46). KEGG and AureoWiki databases were used as references for gene annotation (3, 42). The strain NCTC8325 was used as the reference strain (see Table S3 at https://github.com/clyama/Supplement-for-Yamamoto-and-Kuehn-2026-mBio01598-26 for full table).

### Regulation of EV production by CodY

One of the hypervesiculating mutants we identified is the *codY::tn* mutant. CodY is a repressor of *agr* and is a highly conserved global transcriptional repressor for genes involved in the adaptive response to BCAA starvation (43, 47, 48). CodY senses nutrient availability by direct interaction with metabolite effectors, GTP and BCAAs. Previously, it was shown that CodY integrates nutritional and population density signals through the *agr* quorum-sensing system in *S. aureus* (49). Interestingly, we observed a large number of CodY targets in our vesiculation screen data set (Tables 1 and 2). Of the 173 genes identified to alter vesiculation, 51 (29.5%) also harbor the CodY consensus binding motif within their intergenic and/or coding regions (43–45).

In order to explore the possible role of CodY in *agr*-dependent EV biogenesis, as well as to validate our moderately high-throughput screen data, a larger-volume, quantitative assay using the conventional ultracentrifugation-based vesicle isolation protocol was performed on several mutants, including the *codY::tn* mutant, as well as mutants of both CodY and *agr*-dependent and *agr*-independent genes. Vesicle production, normalized by OD_600_ of each individual culture, was assessed by lipid and protein measurements using FM4-64 and Bradford assays, respectively, and compared to that of the WT parent strain, JE2.

**Fig 4:**
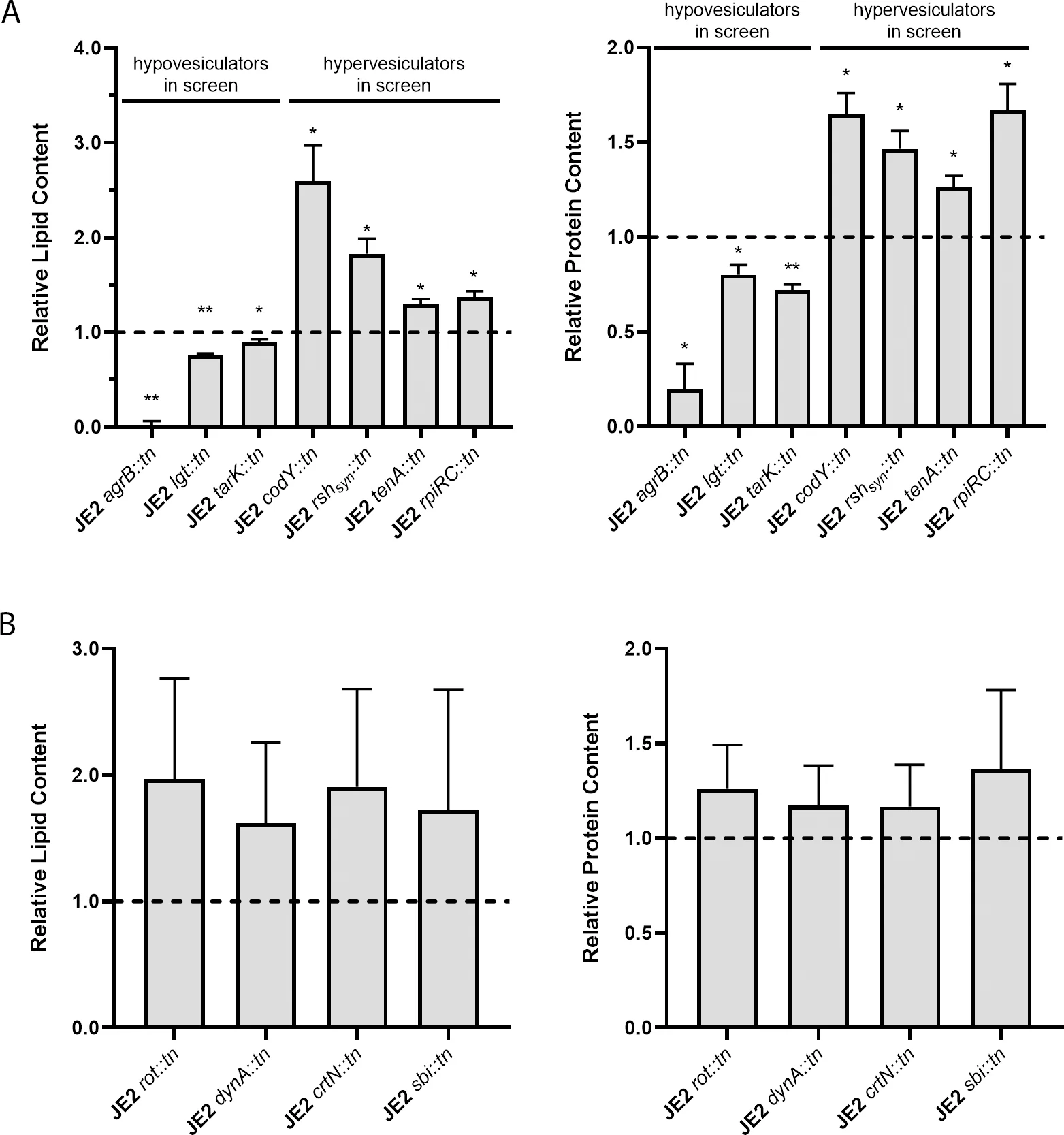
Validation of vesiculation phenotypes of NTML mutants.Vesiculation values for (**A**) EVs purified from vesiculation mutants identified in the screen and (**B**) EVs purified from selected mutants that the screen identified as not having vesiculation phenotypes, compared with the WT JE2 parent strain, represented by the horizontal dashed lines. Values are presented as means from three independent experiments ± SD, normalized to the control. Samples were compared using mixed-effects analysis (**A**) or one-way ANOVA with Dunnett’s multiple-comparison test (**B**). *P < 0.05, **P < 0.01, ***P < 0.001, ****P < 0.0001.

The large-scale validation showed that the c*odY::tn* mutant is indeed hypervesiculating (Fig. 4A). DLS analysis of the EVs produced by the *codY::tn* mutant shows that the size of the vesicles produced by the hypervesiculating *codY::tn* mutant is not different from those produced by the WT strain (Fig. 1C; see Fig. S2 at https://github.com/clyama/Supplement-for-Yamamoto-and-Kuehn-2026-mBio01598-26). As CodY integrates nutritional and population density signals through *agr,* is involved in the biosynthesis and metabolism of BCAAs, the precursors of branched-chain fatty acids (BCFAs), and can influence membrane fluidity, we specifically looked at mutants that affect nutrient sensing and acquisition, membrane properties, or *agr* itself (49, 50). AgrB is part of the *agr* QS system and is involved in the maturation of the quorum-sensing signal, AIP (26). Lgt and TarK affect the membrane properties: the *S. aureus lgt* gene encodes a phosphatidylglycerol-prolipoprotein diacylglyceryl transferase that catalyzes the first step of lipoprotein biosynthesis; thus, the *lgt* mutant is deficient in lipoproteins, a major component of the cellular membrane (51, 52). TarK is regulated by *agr* and influences the length of WTAs, cell wall-associated glycopolymers that are major constituents of the gram-positive cell envelope (53, 54). RelA/SpoT homolog (RSH), TenA, and RpiRC play roles in nutrient sensing and acquisition: RSH is the bifunctional stringent response regulator that regulates the cell’s response to amino acid starvation, specifically the BCAAs leucine and valine through CodY (47, 55). TenA is involved in the biosynthesis of thiamine, an essential cofactor in the central metabolic pathway (56). Finally, RpiRC is a transcriptional regulator of the pentose phosphate pathway (PPP) whose expression is regulated by the *agr* system’s main effector molecule, RNAIII (45, 57).

Figure 4A demonstrates that the moderately high-throughput vesiculation screen developed in this study was successful in identifying the vesiculation phenotypes for the selected mutants, and that nutrient availability, specifically BCAAs and carbohydrates, as well as membrane properties, may be major factors influencing EV production (Fig. 4A).

To further validate the screen results, we selected other mutants that also affect membrane properties and *agr* but were not identified by the screen to have significant changes in their vesiculation. Rot is a cytoplasmic regulator of toxins and extracellular proteases whose expression is *agr*-dependent (26). DynA is involved in membrane remodeling to adapt to membrane stresses, while CrtN catalyzes the second step of staphyloxanthin (STX) biosynthesis, which has been shown to increase membrane rigidity (58–60). Sbi is a highly abundant *agr*-regulated, membrane-associated protein with roles in immune evasion (61). Validation of the vesiculation phenotypes of these mutants using the traditional large-scale method further confirms the accuracy of the developed screen at detecting changes in vesiculation (Fig. 4B).

### Relationship between membrane fluidity and EV production

Previous studies have hinted at a possible relationship between *S. aureus* EV production and membrane fluidity. Schlatterer et al. (22) demonstrated that exogenous addition of synthetic α-PSMs resulted in an increase in membrane fluidity of bacteria, while the *S. aureus psm*α, *psm*β, and *hld* triple mutant had decreased membrane fluidity compared to the WT strain; Wang and colleagues (15) observed increased membrane fluidity in the hypervesiculating *lgt::tn* mutant compared to the WT.

**Fig 5:**
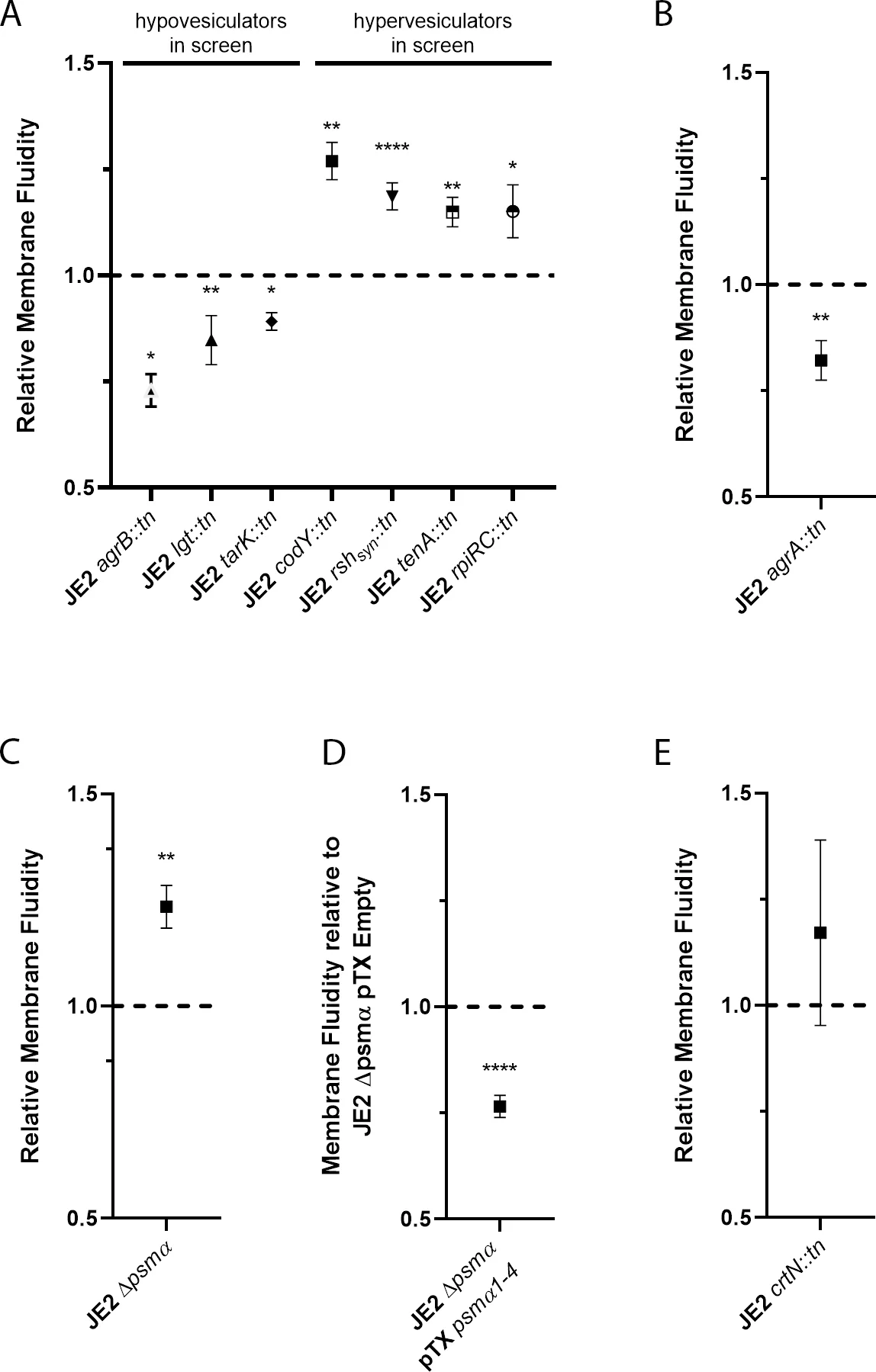
EV biogenesis is regulated by membrane fluidity through CodY. Relative membrane fluidity of *S. aureus* (**A**) vesiculation mutants identified by the screen, (**B**) *agrA::tn*, (**C**) ∆*psm*α*,* (**D**) β∆*psm*α pTX *psm*α*1-4*, and (**E**) *crtN::tn* were measured using pyrenedecanoic acid (PDA) and compared with the JE2 WT (**A, B, C, E**) or JE2 WT pTX empty-vector control (**D**). PDA fluorescence was monitored by excitation at 360 nm and emission at 400 nm (monomer) and 470 nm (excimer). Relative membrane fluidity was calculated as the ratio of excimer to monomer fluorescence and normalized to that of the respective WT control, represented by the horizontal dashed lines. Values are presented as means from three independent experiments ± SD, normalized to the control. Samples were compared using mixed-effects analysis (**A**), one-way ANOVA with Dunnett’s multiple-comparison test (**B and C**), or an unpaired *t*-test (**D and E**). *P < 0.05, **P < 0.01, ***P < 0.001, ****P < 0.0001.

Many gram-positive bacteria, including *S. aureus*, do not produce any unsaturated fatty acids (59). Instead, they synthesize branched-chain fatty acids from BCAAs to incorporate into phospholipids to increase membrane fluidity. Because BCFAs are derived from BCAAs, and CodY is an inhibitor of BCAA biosynthesis and import genes, we wondered if the *codY::tn* mutant had increased membrane fluidity. Additionally, we also assessed the membrane fluidity of the other mutants to determine a relationship between EV biogenesis and membrane fluidity. We used the fluorescent lipophilic pyrene probe, pyrene decanoic acid (PDA), to quantitatively measure membrane fluidity. In all cases we tested, a hypovesiculating phenotype is accompanied by a decrease in membrane fluidity, and a hypervesiculating phenotype is accompanied by an increase in fluidity compared to the WT strain (Fig. 5A through C).

The balance of BCFAs, straight-chain fatty acids, and STX determines the membrane fluidity of *S. aureus* (59). We observed that the strain deficient in STX did not have a change in vesiculation or membrane fluidity compared to the WT (Fig. 4B and 5E). On the other hand, lipidomic analysis of the hypervesiculating JE2 ∆*psm*α mutant revealed an increase in shorter and BCFAs incorporated into their phospholipids compared to the WT JE2 strain, and complementation of the JE2 ∆*psm*α mutant decreased vesiculation and membrane fluidity (Fig. 5D; see Fig. S6 at https://github.com/clyama/Supplement-for-Yamamoto-and-Kuehn-2026-mBio01598-26). Taken together, these results suggest that EV biogenesis may be regulated by membrane fluidity and that the change in membrane fluidity associated with EV production is due to changes in the BCFA content in the membrane, which is dependent on CodY.

### Nutritional stress promotes EV biogenesis

RSH and RpiRC have roles that influence the metabolic state of the cell through the stringent response and the central metabolic pathway, respectively (55, 57). Intriguingly, they are also involved in the *agr* QS system: While the expression of α-PSMs, which we demonstrated to negatively influence EV production, is directly regulated by AgrA, modulation of their expression is dependent on the RSH synthetase activity (25, 26, 62). On the other hand, expression of RpiRC, a transcriptional regulator of the pentose phosphate pathway, is regulated by the *agr* system’s main effector molecule, RNAIII, which we demonstrated to facilitate EV production (Fig. 2) (45, 57).

**Fig 6:**
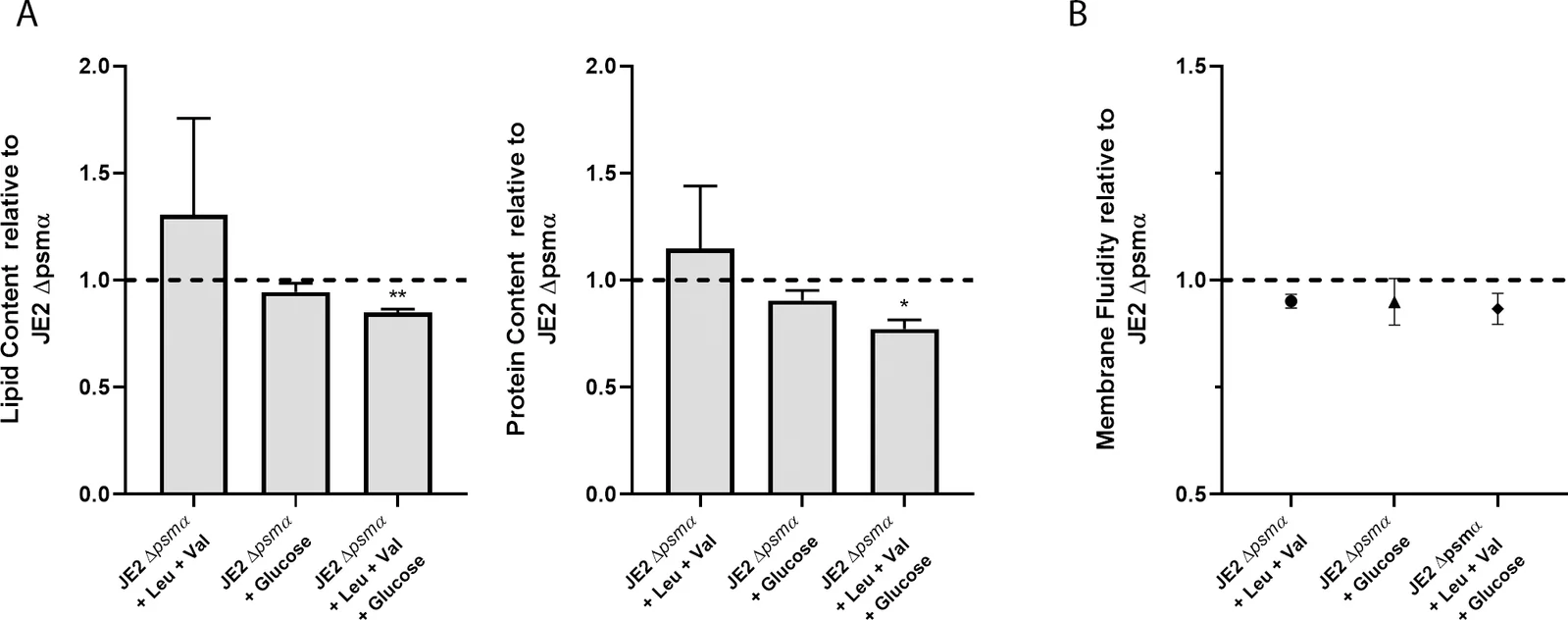
Effects of glucose and BCAA supplementation on EV production of the JE2 Δ*psm*α mutant. (**A**) Supplementation with 455 μM each of leucine and valine and 7 mM glucose reduces EV production in the JE2 Δ*psm*α mutant without supplementation. (**B**) Glucose and BCAA supplementation does not significantly reduce membrane fluidity in the JE2 Δ*psm*α mutant compared with the JE2 Δ*psm*α mutant without supplementation. (**C**) Relative membrane fluidity of supplemented Δ*psm*α mutants compared with the JE2 Δ*psm*α mutant without supplementation. Values are presented as means from three independent experiments ± SD. Samples were compared using one-way ANOVA with Dunnett’s multiple-comparison test. *P < 0.05, **P < 0.01, ***P < 0.001, ****P < 0.0001.

Since *psm*α expression is regulated by the synthetase activity of RSH, and the synthetase activity of RSH is governed by nutrient (i.e., BCAA) availability, and *agr*-dependent EV production also seems to be influenced by the central metabolic pathway, we wondered if the hypervesiculating phenotype of the ∆*psm*α mutant is caused by nutrient limitation. To address this, we supplemented the JE2 ∆*psm*α mutant with the BCAAs valine and leucine (455 µM), glucose (7 mM), or both to determine if increasing nutrient availability can reduce EV production. As shown in Fig. 6A, supplementation of the JE2 ∆*psm*α mutant with BCAAs or glucose alone does not cause a significant change in EV production compared to the JE2 ∆*psm*α mutant, but addition of both significantly decreased EV production by 15.28% ± 0.04% by lipid measure and 19.48% ± 0.23% by protein measure, suggesting that nutrient availability is inversely related to EV production (Fig. 6A).

We did not observe a significant decrease in membrane fluidity despite the observed decrease in EV production when supplementing with BCAAs and glucose (Fig. 6B). The cell membrane is limited to a narrow window of fluidity to remain viable, and the JE2 ∆*psm*α mutant is severely hypervesiculating; it would still be hypervesiculating compared to the WT, even with a 15%–20% decrease in its EV production (Fig. 1B, 5C, and 6A and B). Therefore, it is likely that any changes in membrane fluidity would not be drastic enough to be accurately detected by the fluorescent probe.

**Fig 7:**
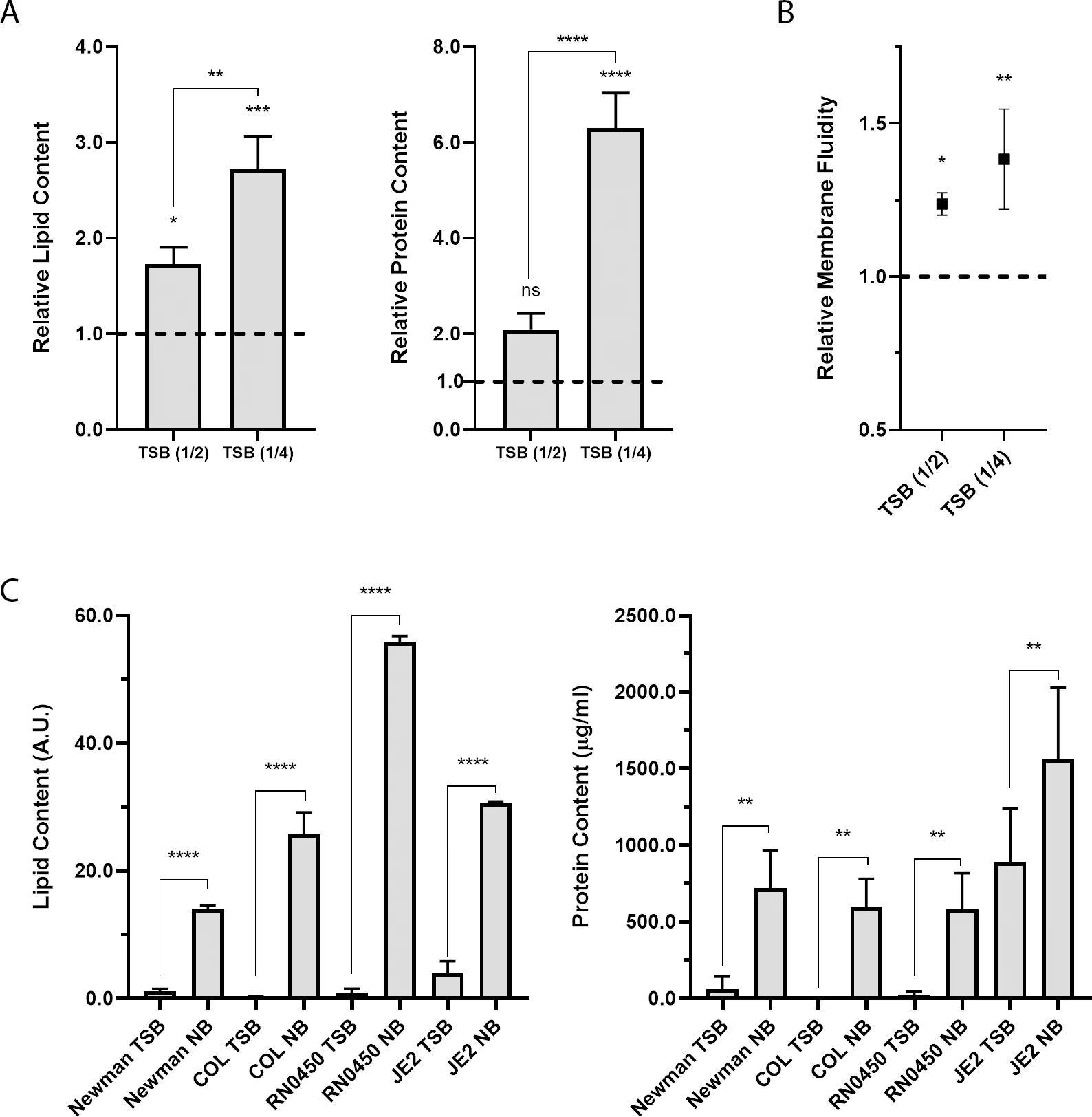
*S. aureus* vesicle production is inversely related to nutrient availability. (**A**) Vesiculation values for EVs purified from JE2 grown for 18 h in 100%, 50%, or 25% TSB. (**B**) Relative membrane fluidity of JE2 grown in 50% and 25% TSB compared with JE2 grown in 100% TSB. (**C**) EVs purified from MRSA strains COL and JE2 and MSSA strains Newman and RN0450 grown for 18 h in rich medium (TSB) or moderately rich medium (NB). Values are presented as means from at least three independent experiments ± SD. Samples were compared using one-way ANOVA with Tukey’s multiple-comparison test (**A and B**) or Sidak’s multiple-comparison test (**C**). *P < 0.05, **P < 0.01, ***P < 0.001, ****P < 0.0001; ns, *P* > 0.05.

To further look into the relationship between nutrient availability and EV production, we isolated vesicles from WT JE2 cells grown in 100%, 50%, or 25% nutrient concentration of TSB while retaining the salt and buffer concentrations. We observed an inverse correlation between EV production and nutrient availability—EV production increased as nutrient concentration decreased (Fig. 7A). With the increase in EV production, we again observed an increase in the fluidity of the membrane (Fig. 7B).

Taken together, these results demonstrate that nutrient availability indeed has an influence on EV production, and nutrient availability is inversely related to EV production. These results also suggest that the strain devoid of α-PSMs is experiencing or responding to nutritional stress or apparent nutritional stress, presumably through the stringent response, causing the increase in EV production.

To determine if the inverse relationship between nutrient availability and EV production is conserved between strains, we grew *S. aureus* JE2, a methicillin-resistant *S. aureus* (MRSA) strain, and several other *S. aureus* MRSA and methicillin-sensitive *S. aureus* strains in rich medium (TSB) or moderately rich growth medium (nutrient broth [NB]) and assessed their vesicle production. Vesicles were isolated from cultures grown overnight (18 h) to ensure all strains had reached stationary phase and to maximize nutrient depletion in the growth medium. We observed that all strains produced more vesicles in the moderately rich growth medium compared to the rich growth medium, demonstrating that the inverse relationship between EV production and nutrient availability is not specific to the JE2 strain (Fig. 7C).

### Effects of other stress on EV production

**Fig 8:**
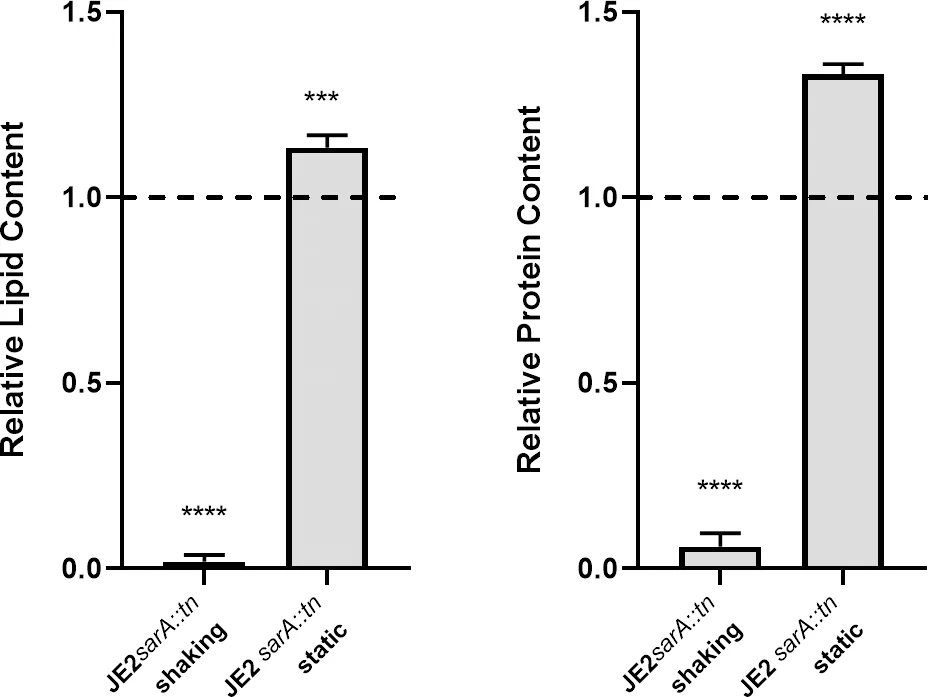
The *sarA::tn* mutant’s vesiculation phenotype is dependent on environmental factors. Vesiculation values of the *sarA::tn* mutant compared to JE2. The *sarA::tn* mutant’s vesiculation phenotype was inconsistent with the screen result when cells were grown in a flask with shaking. Static growth in a beaker phenocopied the screen result. Values are presented as means from three independent experiments ± SD, normalized to that of the wild-type JE2 parent strain grown in a flask with shaking, represented by the horizontal dashed lines. Samples were compared using one-way ANOVA with Dunnett’s multiple-comparison test. *P < 0.05, **P < 0.01, ***P < 0.001, ****P < 0.0001.

We encountered one instance where the screen phenotype appeared to be the complete opposite of the phenotype we observed when vesicles were isolated using the conventional EV isolation protocol. The *sarA* mutant was classified as a hypervesiculator in the screen (Table 1). However, we initially found the *sarA* mutant strain to be severely hypovesiculating compared to the WT strain in our large-scale verification (Fig. 8, shaking). SarA is a member of the SarA family of transcriptional regulators and is a global regulator of virulence factors (63). The *sar* locus contains three promoters, P1, P2, and P3, each capable of producing a transcript encoding SarA. While the transcription of *sarA* from the P1 and P2 promoters is dependent on the housekeeping sigma factor, sigma-A (σ^α^), transcription from the P3 promoter requires the alternative sigma factor, σ^B^, which is activated under various stress conditions (64). Because the time point we chose to assess vesicle production coincides with the maximal expression of the σ^B^-dependent *sarA* transcript, as well as the time point in which σ^B^ is most active, we wondered if the growth conditions of the screen had an effect on EV biogenesis. To test this, we simulated growth in a 96-well microtiter plate in a larger scale by growing the *sarA* mutant in a beaker without any shaking. Compared to the WT strain grown in a flask and with shaking, the *sarA* mutant grown in the microtiter plate-mimicking condition exhibited a hypervesiculating phenotype, just as we observed in the screen (Fig. 8, static). This result suggests that EV biogenesis is highly dependent on environmental factors, and the vesiculation phenotypes of mutants identified in the screen should be verified in the growth conditions of interest. In this case, it appears that SarA is involved in regulating EV production in response to cellular stress and suggests a possible role for EVs in relieving cells from environmental stressors.

**Fig 9:**
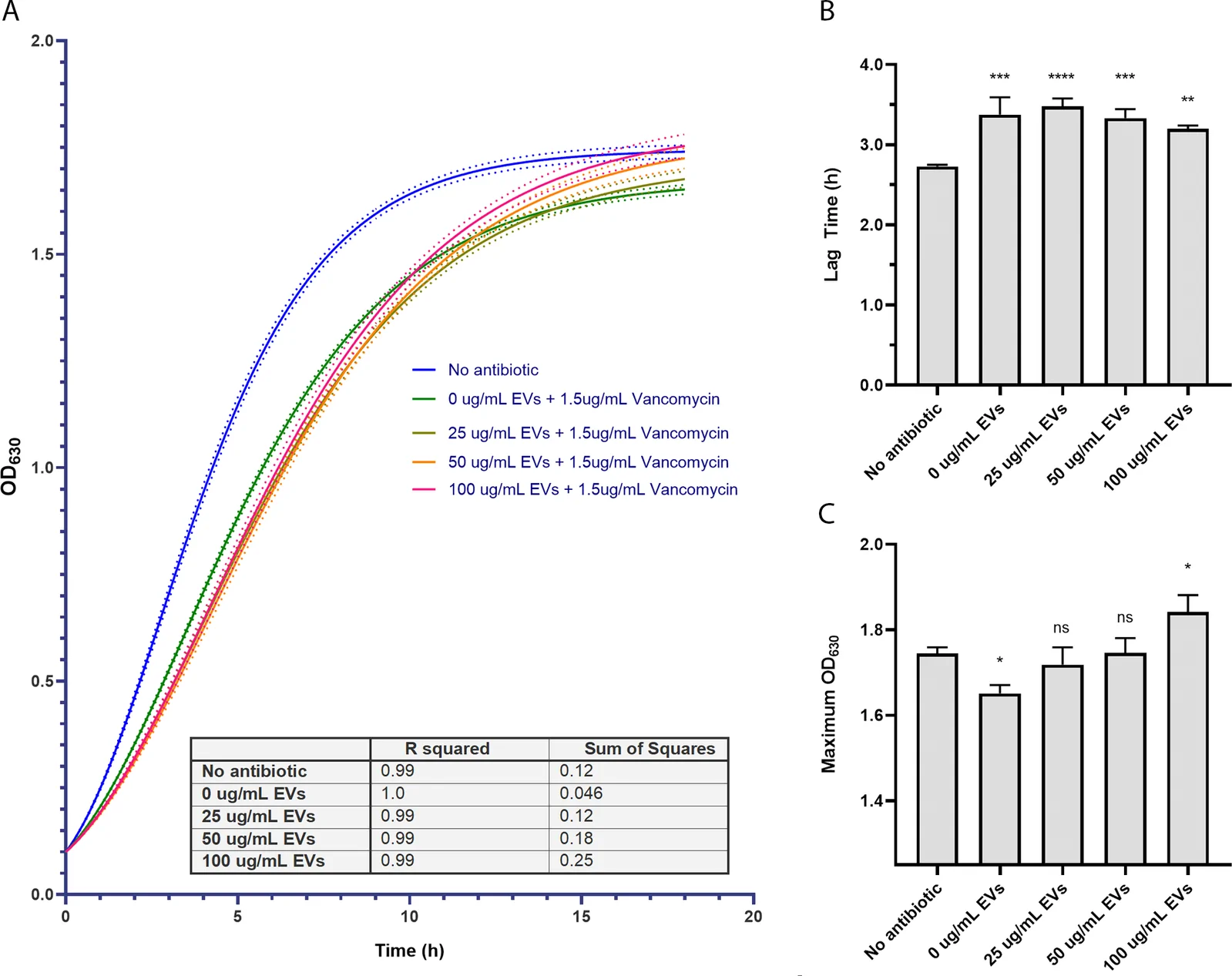
EVs increase tolerance to vancomycin. (**A**) Best fit curves of *S. aureus* JE2 growth in TSB in the presence of 1.5 µg/ml vancomycin and increasing purified EV concentrations determined by a Gompertz least-squares regression model. Dashed lines represent the 95% confidence bands for the best-fit curve. *R*^*2*^ values and sum of squares were determined as measures of goodness of fit. Lag times (**B**) and maximum cell density values (**C**) were derived from best-fit values determined by a Gompertz least squares regression model from three biological replicates. Values are presented as means from three independent experiments ± SD. Samples were compared using one-way ANOVA with Dunnett’s multiple-comparison test. *P < 0.05, **P < 0.01, ***P < 0.001, ****P < 0.0001; ns, *P* > 0.05.

To investigate the potential role of EVs in relieving cells from environmental stressors, we grew *S. aureus* JE2 in a sublethal concentration of vancomycin. Vancomycin is a glycopeptide antibiotic derived from the gram-positive organism *Amycolatopsis orientalis* and is a commonly used antibiotic for treating *S. aureus* infections. We grew JE2 in the presence of 1.5 µg/mL vancomycin and in the presence of varying concentrations (0, 25, 50, and 100 µg/mL) of purified EVs isolated from JE2 overnight cultures grown in TSB. We used Gompertz least squares regression to model the growth, lag time, and maximum culture density. Treatment with the subinhibitory concentration of vancomycin alone increased the lag time of *S. aureus* growth and decreased the maximum density achieved by the culture, as expected (Fig. 9A and B). Interestingly, however, we noted a dose-dependent increase in the maximum culture density when EVs were added in addition to vancomycin. Addition of 25 and 50 µg/mL EVs resulted in maximum cell densities comparable to the no-antibiotic control, with the culture supplemented with 100 µg/mL EVs growing to a higher cell density than the no-antibiotic control (Fig. 9A and C). This suggests that not only does EV production increase, but the presence of EVs can positively affect *S. aureus* survival in conditions of environmental stress.

### Adjusting the screen parameters identifies more hypervesiculating mutants

The *agrA* mutant was previously shown to be hypovesiculating (18, 24). Isolation and quantification of vesicles from the *agrA::tn* mutant, indeed, show that it is a hypovesiculator; however, the *agrA::tn* mutant was not identified in our screen to have a vesiculation phenotype (Fig. 1A). This result drew our attention to the impact of our screen design on the evaluation of MAD values. The MAD values were calculated from values within each specific plate to minimize errors that would arise from day-to-day differences in the fluorescence intensities of the various fluorescent probes used in the screen, based on the key assumption that the average vesiculation value of each plate would be representative of WT vesiculation. Therefore, if a particular plate is skewed to contain more mutants of one vesiculation phenotype, this could exclude some mutants with the opposite phenotype from being identified in the screen. Second, the selected vesiculation cutoff of 2 MADs away from the mean normalized vesiculation value was arbitrarily assigned during the design of the screen. For the *agrA::tn* mutant to be considered a hypovesiculator in our screen, the MAD cutoff would have to be adjusted to 1.2 MADs away from the mean vesiculation value. When we adjusted the stringency to 1.5 and 1.2 MADs away from the mean vesiculation value, we identified an additional 207 and 435 (207 plus 228) mutants, respectively, to have vesiculation phenotypes (see Tables S4 to S7 at https://github.com/clyama/Supplement-for-Yamamoto-and-Kuehn-2026-mBio01598-26).

**Fig 10:**
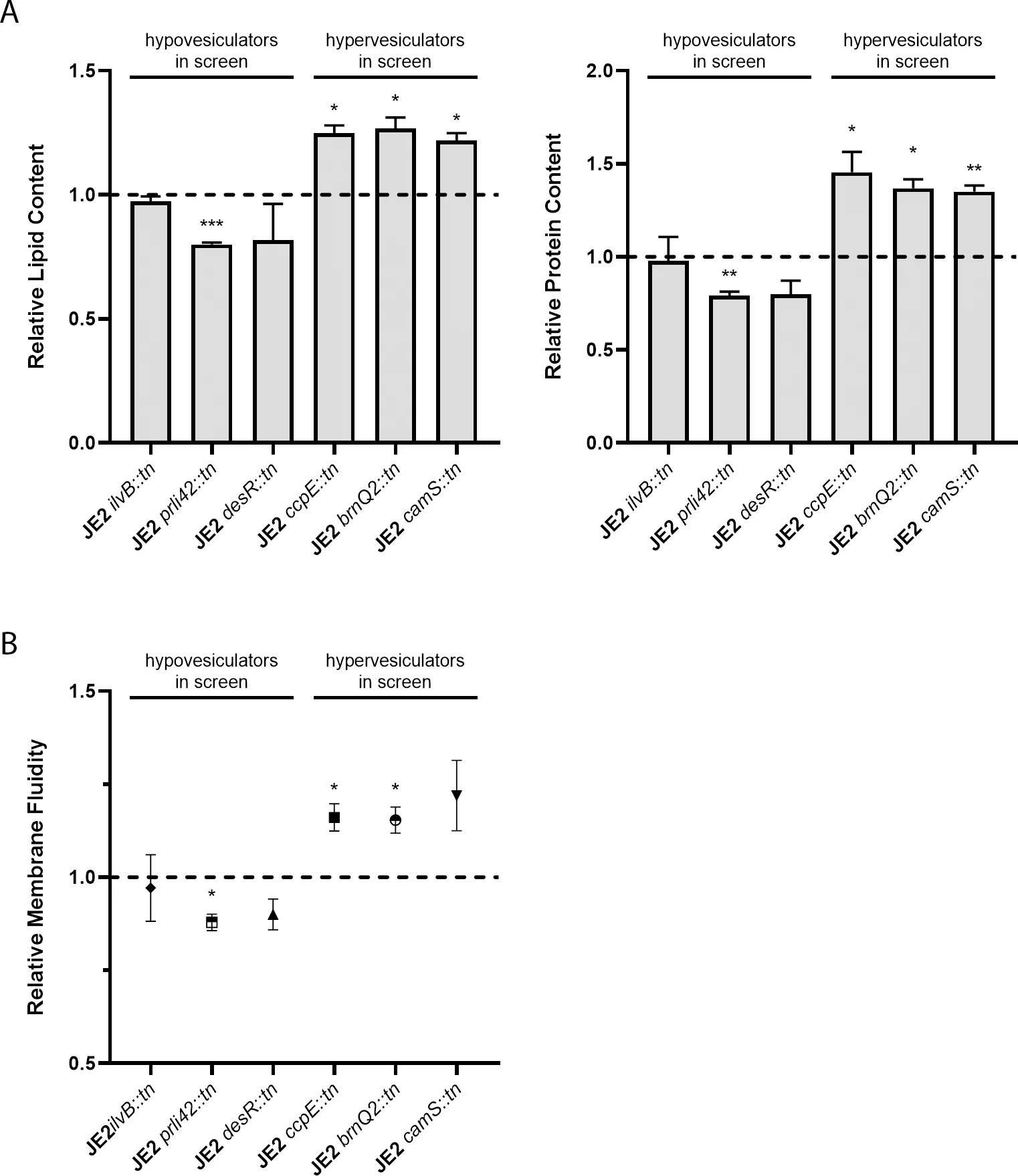
Decreasing stringency of screen parameters still successfully identifies hypervesiculators. (**A**) Lipid and protein contents of EVs purified from mutants were compared to those of the wild-type JE2 parent strain represented by the dashed lines. (**B**) Relative membrane fluidity of mutants was compared to that of the wild type JE2 parent strain, represented by the dashed lines. Values are presented as means from three independent experiments normalized to the control ± SD. Samples were compared using mixed-effects analysis with Dunnett’s multiple-comparison test. *P < 0.05, **P < 0.01, ***P < 0.001, ****P < 0.0001.

We decided to isolate and assess EV production from several mutants identified using these less stringent parameters to determine if these less stringent cutoff parameters can still successfully identify mutants with vesiculation phenotypes. While the less stringent parameters successfully identified hypervesiculators, they were less reliable in identifying hypovesiculators (Fig. 10A and B), suggesting that higher stringency parameters should be used to reliably identify hypovesiculating mutants from this screen.

## DISCUSSION

**Fig 11:**
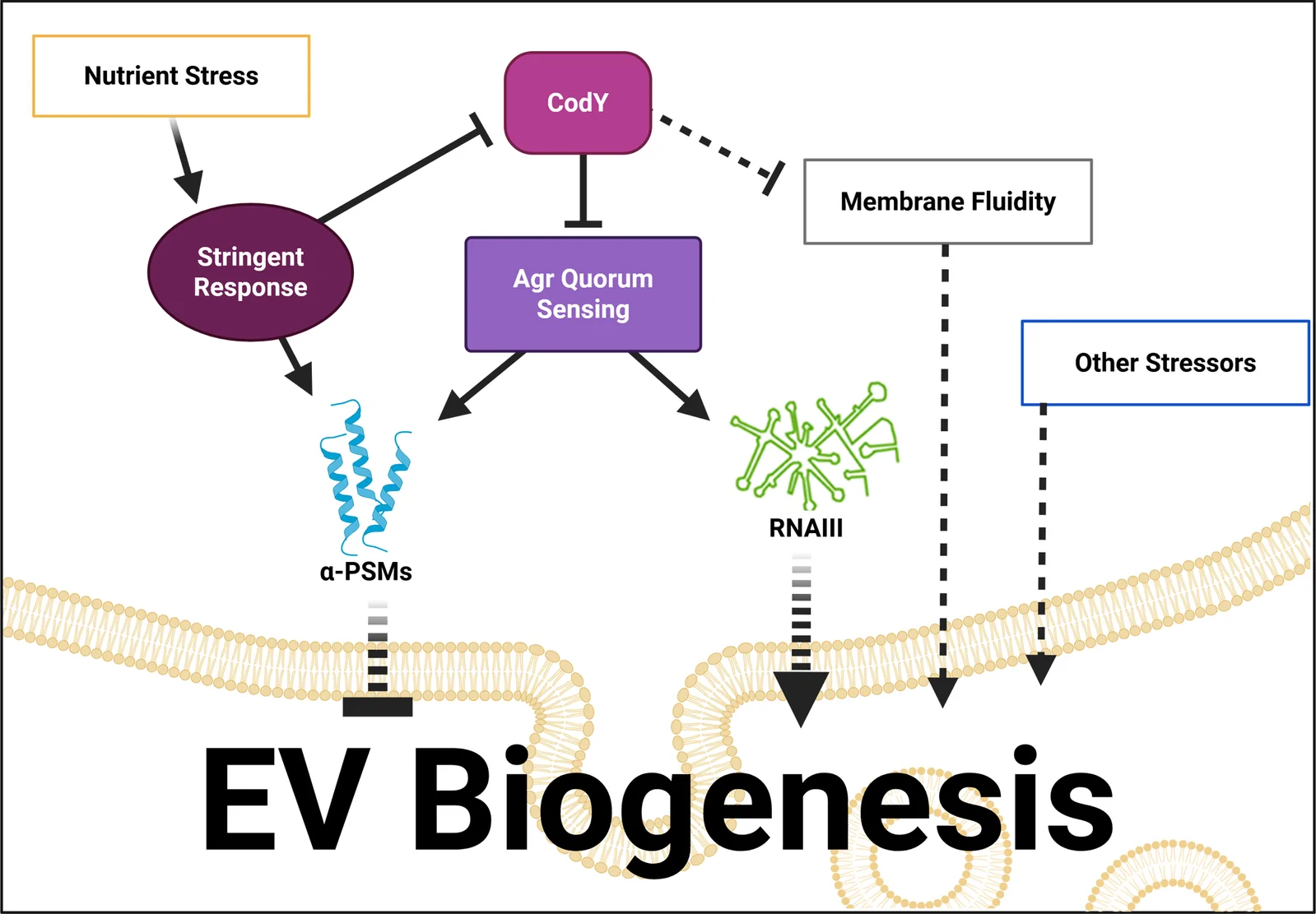
Model for regulation of EV biogenesis in *S. aureus.* Nutrient limitation promotes EV production, presumably by activating the stringent response, which promotes the expression of CodY-regulated genes and activation of the *agr* quorum-sensing system. The *agr* system largely controls EV biogenesis through RNAIII. In addition to AgrA, the stringent response regulator, RSH regulates the expression of α-PSMs, whose effect on EV biogenesis is opposite to and independent of RNAIII. Additionally, membrane fluidity, which can be influenced by CodY through BCAAs, is correlated with EV biogenesis.

To further our understanding of *agr*-dependent EV biogenesis in *S. aureus*, we developed, validated, and utilized an unbiased screen for identifying mutants in the NTML with vesiculation phenotypes. Our data point to CodY as a mediator of *agr*-dependent EV biogenesis and suggest that nutrient limitation is a driver of vesicle production (Fig. 11). Interestingly, early work on OMVs has hinted at a relationship between nutrient limitation and bacterial EV production as early as 1966 (65).

We demonstrate that the CodY-regulated *agr* QS system is a key mediator of EV production and that the AgrA targets, α-PSMs and RNAIII, have opposing contributions to EV biogenesis. Furthermore, our results establish a link between the stringent response and PPP to the quorum-sensing system in regulating vesiculogenesis through α-PSMs and RNAIII. During the preparation of this article, a study was published by Mansour and colleagues (66) demonstrating that the disruption of the stringent response alters the sRNA cargo of EVs in *S. aureus*, providing more evidence in recognizing a role for the stringent response in EV biogenesis.

Overall, our results demonstrate that EV production is a response to cellular stress, including nutritional stress, and is largely regulated and orchestrated by the *agr* quorum-sensing system, possibly through crosstalk with other cellular pathways such as the stringent response through α-PSMs and central metabolism (i.e., PPP) through RNAIII.

Production of vesicles is a universally conserved process, suggesting the existence of a conserved mechanism or principle for EV biogenesis. The stringent response is a conserved bacterial stress response, and recent advances in stringent response research have shown a recurring theme. Several studies have shown ribosome-independent stimulation of the stringent response by direct binding of small regulatory proteins to RSH to control cellular (p)ppGpp levels in both gram-positive and gram-negative bacteria (67–74).

In *S. aureus*, most genes that are activated during the stringent response are regulated through CodY derepression (62). Geiger and colleagues (62) identified 150 (p)ppGpp-dependent genes whose upregulation during the stringent response was dependent on CodY. In contrast, they found only 11 (p)ppGpp-dependent genes that were upregulated during the stringent response independently of CodY; four coded for the α-PSMs and two for the β-phenol soluble modulins (β-PSM1 and β-PSM2, collectively β-PSMs). Similarly, the majority of the genes activated by *agr* are regulated through RNAIII, while genes upregulated by *agr* independently of RNAIII almost exclusively comprise the *psm*α and *psm*β genes (25). Interestingly, *S. aureus* possesses another α-PSM, delta hemolysin (*hld*), whose expression is regulated by RNAIII (21). While the *psm*α locus is conserved in all *S. aureus*, including non-pathogenic strains, *hld* is present mostly in pathogenic strains.

Thus, perhaps α-PSMs act as a regulatory feedback inhibitor of RSH to fine-tune the stringent response to regulate the degree of EV production by *agr* based on nutrient availability through the stringent response. This is interesting and important to address in future studies and may uncover a conserved principle of EV biogenesis in bacteria.

We showed that EV biogenesis directed by the *agr* QS system occurs through RNAIII. RNAIII has been shown to regulate the translation of the PPP regulator *rpiRc*, while RpiRC accumulation downregulates *rnaiii* transcription (45, 57, 75). In the absence of RpiRC, RNAIII transcription is increased, resulting in an increase in virulence factor production and secretion, and thus pathogenicity (76, 77). Thus, it is possible that RNAIII can also regulate the degree of EV production based on the metabolic status of the cell through RpiRC. This is an interesting idea and warrants further investigation.

To our knowledge, ours is the first genome-wide screen to identify genetic determinants of gram-positive EV production. The results of our screen implicate 173 genes influencing vesiculogenesis in *S. aureus*, and, with an appropriate note of caution to investigators for the need to validate phenotypes for individual culture conditions, this will provide a valuable resource for future studies into the mechanism and regulation of EV production.

## MATERIALS AND METHODS

### Bacterial strains and plasmids

*S. aureus* strains were grown in tryptic soy broth (Millipore 22092) at 37°C with shaking at 250 rpm unless stated otherwise. TSB (1/2) and TSB (1/4) contain 50% and 25%, respectively, of casein peptone, soy peptone, and glucose of normal TSB—salt and buffer concentrations were not altered. *E. coli* strains were grown in Luria-Bertani (Millipore VM782285) broth at 37°C with shaking at 250 rpm. Antibiotics were added in the following concentrations when appropriate: *S. aureus*: chloramphenicol (Fisher Scientific, BP904, 10 µg/mL), erythromycin (Sigma, E5389, 10 µg/mL), anhydrotetracycline (Thermo Scientific, J66688.MB, 1 µg/mL), and tetracycline (Sigma, T3258, 10 µg/mL); *E. coli*: chloramphenicol (Fisher Scientific, BP904, 10 µg/mL) and ampicillin (Sigma, A6140, 100 µg/mL). All bacterial strains and plasmids used in this study are listed in Table 4.

**TABLE 4:** Strains and plasmids used in this study

Strain or plasmid	Source or reference
*S. aureus* strains	
JE2 (NTML parent strain)	A gift from Dr. Ken Bayles
NTML	BEI Resources (37)
JE2 (∆*psm*α)	This study
JE2 (∆*rnaiii*)	This study
JE2 (∆*psm*α∆*rnaiii*)	This study
RN4220 (∆*psm*α)	This study
RN4220 (∆*rnaiii*)	This study
RN4220 (∆*psm*α∆*rnaiii*)	This study
RN0450	(78)
Newman	(79)
COL	(80)
Plasmids	
pIMAY	(81)
PCN40	BEI Resources (82)
pTX16	(19)
pTX16 psm1-4	(19)
PCN40 psm1-4	This study

### Isolation of EVs

Overnight *S. aureus* cultures were diluted to OD_600_ = 0.1 with pre-warmed media (150 mL total culture volume) and grown for 5 h. OD_600_ of cultures was measured prior to harvesting by centrifugation (10,000 × *g*, 20 min, 4°C) and filtration through a 0.22 µm PVDF filter (Merck Millipore Germany). Filtrates were then centrifuged at 200,000 × *g* for 3 h at 4°C. Resulting vesicle pellets were gently rinsed before being resuspended in HEPES-NaCl (10 mM HEPES and 150 mM NaCl [pH 7]) buffer and quantified.

### Screen development

To compare the LIVE/DEAD BacLight (Invitrogen L13152) assay with the SYBR Gold/PI viability assay, *S. aureus* JE2 was grown for 5 h to the early stationary/late exponential phase and assigned to be a 100% live culture. Half of the culture was heat-killed for 20 min and assigned to be a 0% live culture. Both cultures were mixed in various ratios and diluted 2- or 10-fold with TSB. For SYBR Gold/propidium iodide (PI), 10 µL SYBR Gold (Invitrogen S11494; 10,000× stock) was mixed with 60 µL PI (20 mM stock, Invitrogen) in 1.0 mL of sterile TE buffer, and 10 µL of the staining mixture was added to each well. The plate was incubated at 37°C in the dark for 15 min. Fluorescence was monitored by excitation at 488 nm and measuring emission at 635 nm for PI and at 535 nm for SYBR Gold (Molecular Devices SpectraMax). The LIVE/DEAD assay was performed per the manufacturer’s protocol (Invitrogen L13152). Fluorescence was monitored by excitation at 488 nm and measuring emission at 635 nm for PI (DEAD) and at 500 nm for SYTO9 (LIVE) (Molecular Devices SpectraMax). To determine FM4-64 sensitivity for detecting EVs, purified *S. aureus* JE2 EVs were diluted with TSB to varying concentrations determined by protein content quantified by the Bradford assay (Bio-Rad). FM4-64 concentrations of 1, 2.5, and 5 μg/mL (Invitrogen, T13320) were used to detect the lipid content of EVs. FM4-64 fluorescence was measured by excitation at 506 nm and measuring emission at 750 nm (Molecular Devices SpectraMax). JE2 growth was assessed by diluting JE2 overnight culture 1:40 with pre-warmed TSB into a 96-well flat-bottom microtiter plate. OD_630_ was monitored in a BioTek ELx808 microplate reader (Agilent) with continuous shaking at 37°C. The first two rows and columns of the 96-well microtiter plate were filled with water to minimize evaporation, and each biological replicate was performed with at least three technical replicates.

### Moderately high-throughput vesiculation screen

Overnight NTML strain cultures were diluted 1:40 with pre-warmed media and were grown for 5 h in 96-well microtiter plates enclosed in a humidity chamber at 37°C with shaking (250 rpm). A volume of 20 µL from each culture was diluted 10× in TE buffer (10 mM Tris-HCl and 1 mM EDTA [pH 8]) in a black, clear-bottom 96-well microtiter plate before OD_600_ was measured. Cell viability was measured from the 10× diluted cells as described previously (41), replacing SYBR Green I with SYBR Gold (Invitrogen, S11494). Briefly, SYBR Gold and propidium iodide (Invitrogen, P1304MP) were used for double staining of nucleic acids. Ten microliters of SYBR Gold (10,000× stock) was mixed with 60 µL of PI (20 mM stock) in 1.0 mL of sterile TE buffer, and 10 µL of the staining mixture was added to each well. The plate was incubated at 37°C in the dark for 15 min. Fluorescence was monitored by excitation at 488 nm and measuring emission at 535 nm (SYBR Gold) and 635 nm (PI) (Molecular Devices SpectraMax). Viability was calculated as a ratio of SYBR/Gold/PI fluorescence. The remaining cultures were sterile-filtered through 96-well, 0.45 μm, PVDF filter plates (Millipore, MSHVS4510) into 96-well black clear-bottom microtiter plates. Vesiculation was measured by incubation of the filtered culture medium with 2.5 µg/mL FM4-64 (Invitrogen, T13320) in the dark at 37°C for 30 min. FM4-64 fluorescence was measured in a black, clear-bottom 96-well microtiter plate by excitation at 506 nm and measuring emission at 750 nm (Molecular Devices SpectraMax). FM4-64 fluorescence was then normalized to OD_600_ of culture at the time of harvest (Molecular Devices SpectraMax). The parameters used to define significant hypo- or hypervesiculation phenotypes are as follows: (i) cell viability value (SYBR Gold/PI) is more than 2 mean average deviations below the mean viability value of the strains within the plate; (ii) vesiculation value (FM4-64) normalized to growth (OD_600_) is over 2 MADs from the mean normalized vesiculation value (FM4-64/OD_600_) of the strains within the plate. MAD was calculated using the formula below, where *x*_*i*_ is the normalized vesiculation value of each mutant, µ is the mean of all the normalized vesiculation values, and *N* is the total number of mutants. $MAD=\;\frac{\sum \;|x_{i}-\;\mu |}{N}.$

### Isolation of EVs for FM4-64 sensitivity assay, vancomycin tolerance assay, and EV yield in TSB and NB comparison

EV isolation was performed as previously described (4). Overnight *S. aureus* cultures grown in TSB or NB (0.5% peptone and 0.3% beef extract [pH 7]) were diluted 1:1,000 in TSB or NB. Cultures were grown for 18 h at 37°C, with shaking (250 rpm) before centrifugation (10,000 × *g* for 20 min at 4°C). The cell-free supernatant was filtered through a 0.45-µm-pore-size PVDF filter (Merck Millipore, Germany). Filtered supernatants were concentrated by ultrafiltration using a tangential flow system (Cole-Parmer MasterFlex) with a 100 kDa hollow-fiber membrane (Pall Corporation), followed by filtration through a 0.22 µm PVDF filter (Merck Millipore Germany). Filtrates were then subjected to ultracentrifugation at 200,000 × *g* for 3 h at 4°C. Pelleted vesicles were resuspended in a small volume of HEPES-NaCl buffer before being subjected to density gradient centrifugation.

### Density gradient centrifugation

Isolated EVs were adjusted to 50% (vol/vol) OptiPrep (Sigma) solution and layered at the bottom of 13.2 mL ultracentrifuge tubes. Samples were then overlaid with a 40% OptiPrep layer and a 10% OptiPrep layer. The tubes were ultracentrifuged at 130,000 × *g* for 16–18 h at 4°C, and 2 mL fractions were collected from the top of the density gradient. The OptiPrep solution was removed by washing each fraction with HEPES-NaCl buffer and ultracentrifugation at 200,000 × *g* for 3 h at 4°C. Each fraction was resuspended in HEPES-NaCl buffer and assayed for protein concentration and lipid content using a Bradford protein assay (Bio-Rad) and 5 µg/mL of FM4-64 dye (Invitrogen, T13320), respectively. EV fractions containing high proteolipid content were combined and subjected to another round of ultracentrifugation (200,000× *g* for 1 h at 4°C). Resulting vesicle pellets were resuspended in 1 mL of HEPES-NaCl buffer before being quantified.

### Quantification of EVs

For the quantification of lipid content, EVs were incubated with 5 µg/mL of FM4-64 (Invitrogen, T13320) in the dark at 37°C for 30 min. FM4-64 fluorescence was measured in a black, clear-bottom 96-well microtiter plate by excitation at 506 nm and measuring emission at 750 nm (Molecular Devices SpectraMax). FM4-64 fluorescence was then normalized to OD_600_ of culture at the time of harvest. Protein content of the vesicles was quantified by a Bradford assay (Bio-Rad). EV diameters and size distribution were evaluated and generated using dynamic light scattering (Wyatt Dynapro Plate Reader III) and the DYNAMICS software version 8.2.0.293 (Wyatt Technology).

### Vancomycin tolerance assay

*S. aureus* JE2 overnight culture was diluted 1:40 with pre-warmed TSB supplemented with 1.5 µg/mL vancomycin (Sigma, 94747) and increasing total protein concentrations of *S. aureus* JE2 EVs isolated from 18 h cultures grown in TSB. Cultures were grown in a 96-well microtiter plate, and growth was monitored by OD_630_ in a BioTek ELx808 microplate reader (Agilent) for 18 h with continuous shaking at 37°C. The first two rows and columns of the 96-well microtiter plate were filled with water to minimize evaporation, and each biological replicate was performed with at least three technical replicates.

### Membrane fluidity

*S. aureus* cultures were normalized to OD_600_ = 1. Cells from 1 mL of OD_600_ = 1 cultures were resuspended in 5 µg/mL of pyrenedecanoic acid (Abcam, ab274309) and 0.08% Pluronic F-127 (Sigma, P2443) in phosphate-buffered saline (PBS) and stained in the dark for 30 min at room temperature with gentle rocking. Cells were rinsed with PBS twice and resuspended in PBS before transferring to a black, clear-bottom 96-well microtiter plate. PDA fluorescence was monitored by excitation at 360 nm and measuring emission at 400 nm (monomer) and 470 nm (excimer) (Molecular Devices SpectraMax). Relative membrane fluidity was calculated as a ratio of excimer to monomer fluorescence.

### DNA manipulation

The JE2 ∆*psm*α, JE2 ∆*rnaii*, and JE2 ∆*psm*α∆*rnaiii* strains were generated by allelic exchange as described previously (81). All deletion strains were confirmed by analytical PCR with genomic DNA. α-PSM complementation was done using the α-PSM-overexpressing plasmid pTx_∆16_ psmα (19) or by PCN40 psmα generated here using the PCN40 expression plasmid constructed by Charpentier and colleagues (82). All *S. aureus* transformation was performed by electroporation. Preparation of electrocompetent *S. aureus* and electroporation were performed as described by Schenk and Ladagga (83). All primers used in this study are detailed in Table 5.

**TABLE 5:** PCR primers used in this study

Purpose	Name	Sequence (5′–3′)
pIMAY		
For RNAIII deletion	Upstream 5′F	GAATTGGAGCTCCACCGCGGTGGCGGCCGCCCGATTGTTTTTGGAGATAGTTCTAAAAATG
	Upstream 3′R	CTAGATCACAGAGATGTGATGGAAAATAGGAGACCCGCCGTTAAAATATATGAATAAG
	Downstream 5′F	GACTTATTCATATATTTTAACGGCGGGTCTCCTATTTTCCATCACATCTCTGTGATCTAG
	Downstream 3′R	GAGGTCGACGGTATCGATAAGCTTGATATCGTGTTAATTCTTTTGGTACTTCAACTTCATC
For α-PSM deletion	Upstream 5′F	GAATTGGAGCTCCACCGCGGTGGCGGCCGGGCTAGCACAATACAATCACGTAG
	Upstream 3′R	GAAGTTAACTCATAAGCAAAGGAGGTAATCGAATACTTAAAATTCTCAGGCCACTATAC
	Downstream 5′F	GGTATAGTGGCCTGAGAATTTTAAGTATTCGATTACCTCCTTTGCTTATGAGTTAAC
	Downstream 3′R	GAGGTCGACGGTATCGATAAGCTTGATATCGATAATTTCGCAGACTCACGTGG
Verification of loss of plasmid	Forward	TACATGTCAAGAATAAACTGCCAAAGC
	Reverse	AATACCTGTGACGGAAGATCACTTCG
Deletion verification	psmα Forward	GGCAAATTAGACCAGCACGA
	psmα Reverse	GACCTAAGACATGTTGACCATG
	RNAIII forward	GGCCAGCTGATTAATGAATACG
	RNAIII reverse	GCTGCAGGAGCATATACTGAG
PCN40		
psmα complementation	psmα Forward	AGTCCTGCAGGCAAAGGAGGTAATCTTAATGGGTATC
	psmα Reverse	AGTCGGATCCCGCTTAAATTATTTTGCGAAAATGTCG
PCN40 insert verification	Forward	GATTTATAAAAATTACAACTGTAATATCGGAGGGT
	Reverse	GGCGATTAAGTTGGGTAACGC

### Gene ontology analysis and gene annotations

Gene Ontology analysis was performed using the ShinyGO version 0.85 database (46). KEGG and AureoWiki databases were used as references for gene annotation (3, 42).

### Statistics

Statistical analyses were performed using GraphPad Prism version 8.0.2 for Windows (GraphPad Software, San Diego, CA, USA, www.graphpad.com).

## Data Availability

Supplemental material, including supplemental figures and tables, is available at https://github.com/clyama/Supplement-for-Yamamoto-and-Kuehn-2026-mBio01598-26.
